# Boosting Tetracycline Degradation with an S-Scheme Heterojunction of N-Doped Carbon Quantum Dots-Decorated TiO_2_

**DOI:** 10.1021/acsomega.3c03532

**Published:** 2023-07-12

**Authors:** Melike Karaca, Zafer Eroğlu, Özkan Açışlı, Önder Metin, Semra Karaca

**Affiliations:** † Department of Chemistry, Faculty of Science, Atatürk University, 25240 Erzurum, Turkey; ‡ Department of Chemistry, College of Sciences, Koç University, Sarıyer, 34450 Istanbul, Turkey; § Koç University Surface Science and Technology Center (KUYTAM), Sarıyer, 34450 Istanbul, Turkey

## Abstract

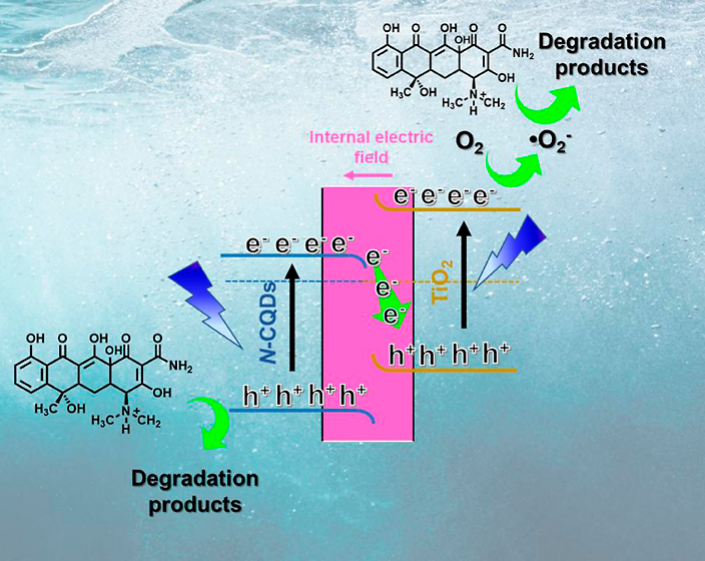

N-doped carbon quantum dots (N-CQDs) derived from the *Rumex crispus* L. plant were incorporated into TiO_2_ via a facile hydrothermal method. As-prepared materials were characterized and used in the photocatalytic tetracycline (TC) degradation under UVA light irradiation by examining several operational parameters involving the N-CQDs amount, initial TC concentration, pH, and photocatalytic reaction time. XRD analysis revealed the conversion of the rutile phase to the anatase phase after the incorporation of N-CQDs into the TiO_2_ structure. The results revealed that the N-CQDs/TiO_2_ photocatalysts demonstrated the highest efficiency in TC degradation compared to other processes of adsorption, photolysis (UVA), and photocatalysis with TiO_2_ (TiO_2_/UVA). Under optimized conditions, 10 mg/L TC at pH 5.15 with 0.2 g/L N-CQDs/TiO_2_ catalyst showed 97.7% photocatalytic degradation for 120 min under UVA irradiation. The formation of an S-scheme heterojunction between N-CQDs and TiO_2_ provided enhanced charge separation and strong redox capability, causing significant improvement in the photocatalytic performance of N-CQDs/TiO_2_. Trapping experiments showed that O_2_^•–^ and h^+^ are the predominant reactive species for the TC elimination in an aqueous solution.

## 1 Introduction

The uncontrolled release of active pharmaceutical ingredients, such as beta-blockers, antiphlogistics, or antibiotics, into the aqueous environment by various means threatens both the ecosystem and human health by inducing the enhancement of bacterial drug resistance.^1−3^ Tetracycline hydrochloride (TC) is one of the most widely used antibiotics for infectious diseases in human health, livestock, and fish farming because it has a broad spectrum of antibacterial properties and remissible side effects, along with its low cost compared to other available options.^3,4^ However, since most of the TC molecules cannot be exactly decomposed and metabolized in living things, they are excreted directly without any change in their structure into the receiving environments by stool and urine.^4,5^ Considering the diminishing of water resources due to drought, it is vital to develop an effective treatment method to remove drug residues from aqueous environments. For this reason, this issue has recently become an important research topic that scientists have been working on intensely. There are several established treatment techniques, such as biological, physical, and chemical processes, that are widely applied to remove antibiotics from wastewater, but traditional treatment techniques are not effective in TC removal due to their low variability and biodegradability.^6,7^ Semiconductor-based photocatalytic oxidation is a green and sustainable technology with characteristics of strong oxidation activity, low energy consumption, operability at room temperature, and ambient pressure, which is used to remove TC and other organic contaminants in water.^6,8,9^ In a photocatalytic oxidation process, parameters such as the harvesting of sunlight in the UV–vis–NIR range, good separation, and transport of the photoproduced e^–^/h^+^ pairs, and the construction of active reaction centers on the surface for redox reactions play a critical role.^10^ In this context, the development of an efficient photocatalytic oxidation process with the design of a photocatalyst with high activity, simple and inexpensive preparation, convincing electron–hole separation, and addition to prepared from natural sources for a sustainable environment is a very important task.

Titanium oxide (TiO_2_), featuring many advantageous properties such as being environmentally friendly, chemically stable, abundant on earth, non-toxicity, and low cost, has been a trend in photocatalytic applications until today.^11,12^ Unfortunately, the relatively wide band gap of TiO_2_ (3.0–3.2 eV) markedly limits its photocatalytic applications. This situation causes problems such as weakening of charge separation, low visible light harvesting, and loss of radiation energy or conversion into heat when recombination occurs.^13^ By managing the band gap of the photocatalyst, tuning its chemical composition, modifying its surface, and designing or assembling hybrid materials, these problems can be overcome, and the activity of the photocatalyst can be increased.^13^

Recently, carbon quantum dots (CQDs) with a size less than 10 nm have received a lot of attention in photocatalytic applications due to their inimitable properties such as strong chemical inertness, low toxicity, low production cost, high chemical stability, and wavelength-dependent luminescence emission.^12,14,15^ It has been reported that CQDs are quite successful in the enhancement of the photocatalytic activity of hybrid materials.^16^ The perfect electron transfer/reservoir properties of CQDs have a high contribution to the increase of photocatalytic efficiency.^12,14,17^ However, research on finding suitable methods to achieve desired photocatalytic activity with CQDs-based materials is still ongoing, and the relationship between the structure and activity of CQDs needs to be examined in detail.^8,18^ Recently, it was reported that carbon materials obtained by incorporating nitrogen into the CQDs structure can effectively promote charge delocalization, decrease the work function of carbon, enhance the photoluminescence performance, as well as provide access to tunable electronic and optical properties.^12,19^ Based on the above discussion, an improvement in photocatalytic activity is expected when N-CQDs are synthesized and combined with semiconductors. On the other hand, in these studies, some improvised mechanisms have been proposed considering the positive contribution and spectral and electron donor/acceptor properties of CQDs for the improved photocatalytic oxidation of organic pollutants. Therefore, a mechanism exhibiting the emergence of excellent photocatalytic activity of the CQDs/TiO_2_ nanocatalyst needs to be studied and revealed in detail.

Herein, we report the fabrication of N-CQDs/TiO_2_ heterojunction photocatalysts for the photocatalytic oxidation of TC in water under UVA illumination. The N-CQDs/TiO_2_ heterojunction photocatalyst was prepared via a green and economic hydrothermal procedure using *Rumex crispus* L. as a carbon source. Compared to the state-of-the-art methods, hydrothermal carbonization method exhibits appealing features such as low production costs, high efficiency, non-toxicity, and environmental friendliness.^15^ The physicochemical properties of the photocatalysts were investigated using advanced analytical techniques. The effect of different parameters involving catalyst amount, TC concentration, and pH level on the photodegradation performance of N-CQDs/TiO_2_ photocatalyst was revealed. Based on trapping studies, a possible mechanism of TC photodegradation was also proposed. The direction of charge transfer at the interfaces of N-CQDs and TiO_2_ is determined by their work function values, which control the direction of charge flow. The formation of an S-scheme heterojunction between the components of the N-CQDs/TiO_2_ photocatalyst promotes charge separation and transfer, resulting in superior photocatalytic activity. This charge carrier redistribution process occurs at the heterogeneous interface through an S-scheme mechanism.

## 2 Experimental Section

### 2.1 Synthesis of N-CQDs

0.5 g of *R. crispus* L. was added to 25 mL of distilled water. Next, 0.3 g of urea and 15 mL of concentrated HCl were added to the aqueous solution of *R. crispus* L. The resultant mixture was transferred to a 100 mL Teflon lined stainless steel autoclave and kept in a temperature-controlled oven at 150 °C for 6 h. Next, the autoclave was cooled to ambient temperature. A pale yellowish solution was obtained and filtered through a 0.45 μm membrane filter to eliminate unreacted particles and centrifuged 10 min at 9000 rpm. Finally, the purified N-CQDs were collected in a clean glass bottle and stored at 5 °C for use in experiments.^20^

### 2.2 Synthesis of TiO_2_

The modified version of the well-known procedures were used for the synthesis of TiO_2_ by our group. Further information on the steps can be found in the Supporting Information.

### 2.3 Synthesis of N-CQDs/TiO_2_

**Figure 1:**
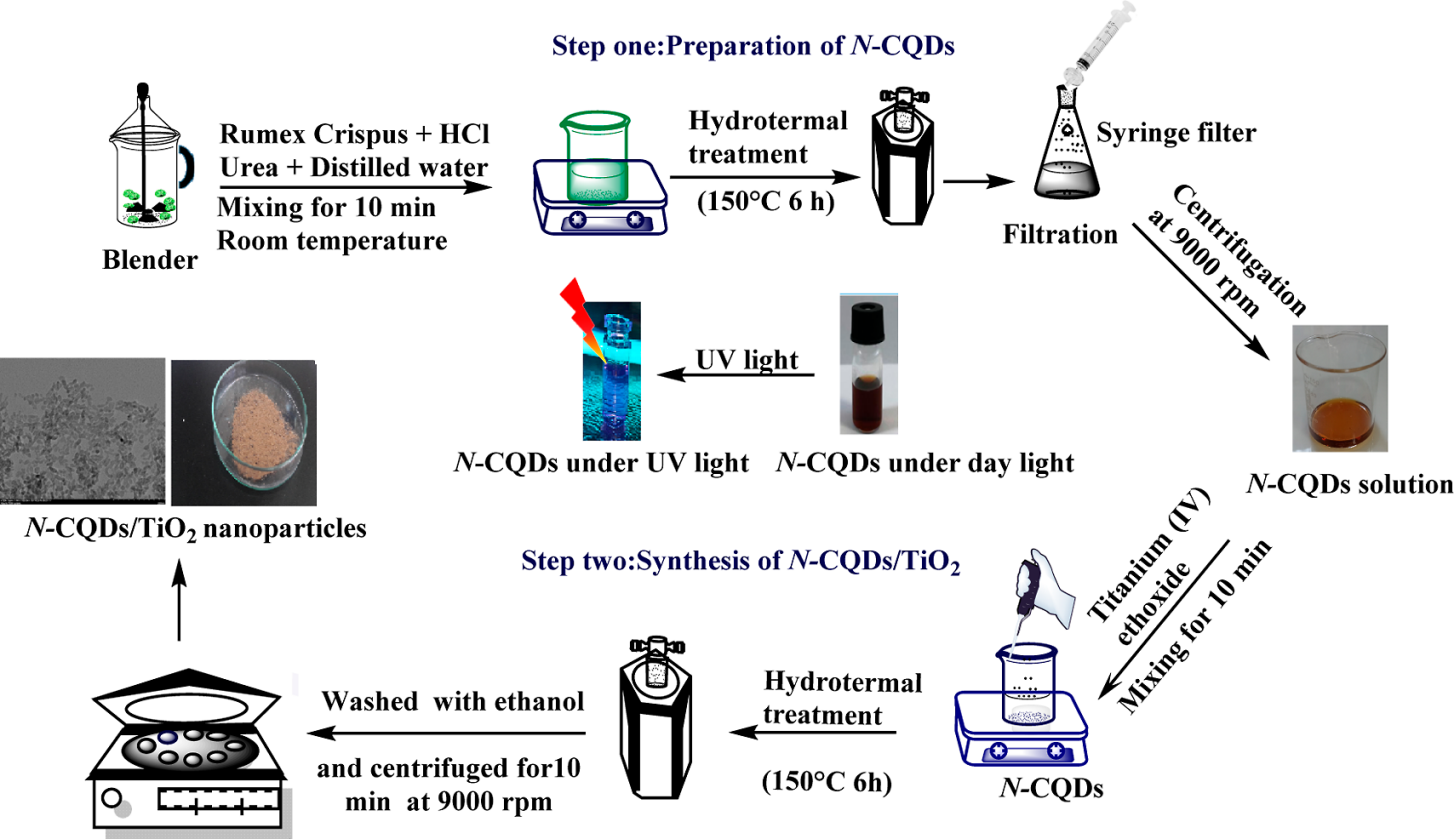
Experimental flow chart for the preparation of N-CQDs/TiO_2_ nanoparticles.

The synthesis of the N-CQDs/TiO_2_ catalyst was carried out in situ by following the flow chart depicted in Figure 1. For this purpose, 10 mL of N-CQDs solution, which was prepared with 0.5 g of *R. crispus* L. as described above, was mixed with 40 mL of water and 0.3 g of urea, and the mixture was stirred for 10 min. 1.6 mL of titanium(IV) ethoxide was added dropwise to this mixture and stirred for an additional 10 min. The prepared mixture was taken to a Teflon-equipped stainless-steel autoclave with an internal volume of 100 mL. The autoclave was placed in a temperature-controlled muffle furnace, heated to 150 °C, and kept at this temperature for 6 h. After the autoclave had cooled down to ambient temperature, the lid was opened, and the reacted suspension was centrifuged at 9000 rpm for 10 min and dried in an oven at 80 °C for 7–12 h.^20^ The resulting N-CQDs/TiO_2_ catalyst was stored in closed containers for use in experiments. The preparation of the catalyst is schematically shown in Figure 1.

## 3 Results and Discussion

### 3.1 Catalyst Characterization

N-CQDs/TiO_2_ photocatalysts were prepared by using a hydrothermal method following the experimental steps shown in the flow chart (Figure 1). We fabricated N-CQDs by one-step hydrothermal treatment of *R. crispus* L. in the presence of water, urea, and concentrated HCl at 150 °C for 6 h. Likewise, pure TiO_2_ nanoparticles were prepared by using the same procedure without N-CQDs in the medium. The production of N-CQDs was confirmed by XRD, TEM, FT-IR, and HR-TEM analyses.

**Figure 2:**
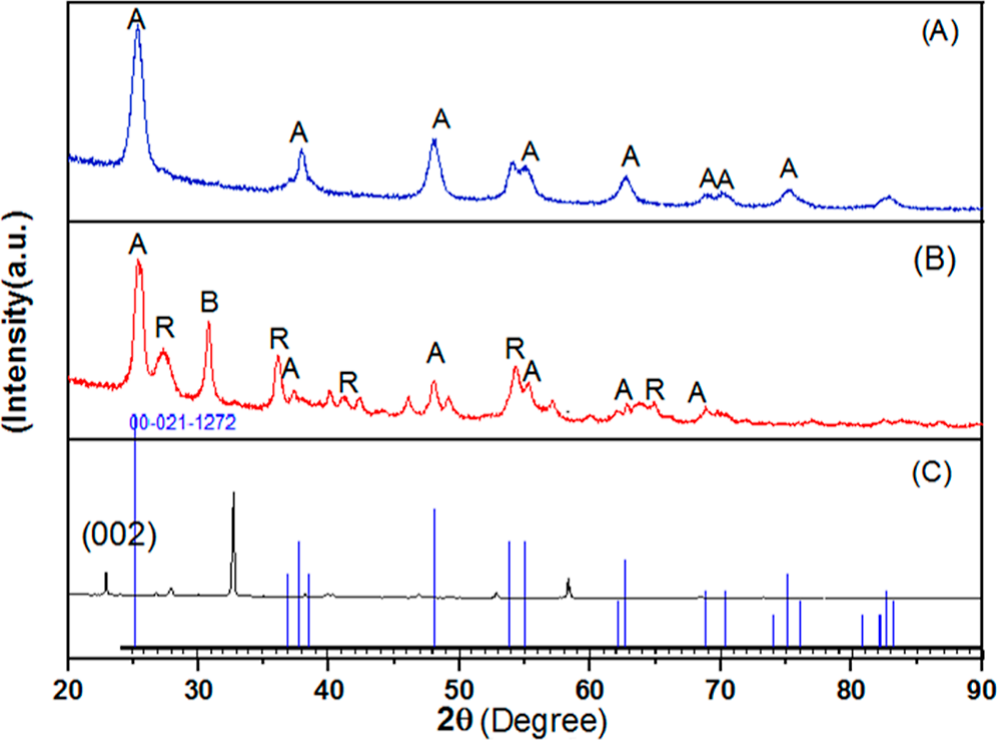
XRD patterns of (a) N-CQDs/TiO_2_ nanocomposites, (b) bare TiO_2_, and (c) N-CQDs.

Crystal structure analysis of pristine N-CQDs, TiO_2_, and N-CQDs/TiO_2_ nanocomposites was performed with powder XRD, and the recorded diffractograms are illustrated in Figure 2. The studies to date have generally revealed that the photocatalytic performance of the catalyst is mainly dependent on crystallinity, the ability to harvest light, and the recombination of photoinduced charge carriers. Powder XRD is an important and rapid analytical method often used in research to identify the phases of any crystalline material.^13^ The XRD pattern of synthesized N-CQDs shows a characteristic sharp diffraction peak located at 2θ = 22.91° (Figure 2), attributing it to the (002) planes of graphitic carbon with sp^2^ hybridization.^21,22^ The *d*-spacing of the (002) peak at 2θ = 22.91° is calculated to be 0.395 nm, which is larger than that of the graphitic interlayer spacing (0.32 nm), revealing that the prepared sample is not bulk graphitic carbon. The change in interplanar distance may have been caused by the formation of more oxygen-containing and amine groups, which were formed by hydrothermal reactions during the hydrothermal preparation of N-CQDs on the surface and edges of the N-CQDs.^22,23^ The two peaks at 2θ = 26.75 and 27.93° appear, which are readily indexed to the graphene and graphene oxide structures.^24^ The pronounced diffraction peak at 2θ = 30.3° for N-CQDs can be attributed to the (200) disordered graphite-like structures.^25^ Additionally, the remarkable peaks at 2θ = 32.6 and 32.68° can be ascribed to disordered graphite-like species of N-CQDs.^26^ The other characteristic peaks of the N-CQDs were detected at 2θ = 40.38 and 58.29°, which are readily assigned to the (100) and (103) reflections, respectively. The first peak indicates the presence of graphitic sp^2^ carbon clusters,^27^ while the second peak implies a diamond-like carbon structure of sp^3^ hybridization.^28^ The obtained results are in very good agreement with the studies in which CQDs were synthesized from coconut coir using the hydrothermal method by Chauhan et al.^29^ and from polymers and organic solvents via the immersion method by Das et al.^30^ From the XRD pattern of bare TiO_2_, the existence of both anatase and rutile forms of TiO_2_ is clearly evident. Similar results have been reported by Alamelu et al.^31^ (Figure 2). The peaks appearing at 2θ = 25.3, 37.36, 48.03, 55.19, 62.78, and 68.94° are attributed to (101), (103), (200), (105), (204), and (116) reflection planes of anatase phase (JCPDS 21-1272), respectively.^13^ The peaks located at 2θ = 27.27, 36.15, 41.43, 54.38, and 64.06° are well indexed to (110), (101), (111), (211), and (002) crystal planes of the rutile phase, respectively (JCPDS Card no. 21-1276).^32^ In addition, the main peak displayed at an angle 30.78° also indicates the peak belonging to brookite(111) (JCPDS no. 84-1750).^33^ The XRD pattern of N-CQDs/TiO_2_ nanocomposites displays only the reflection planes of the anatase phase of TiO_2_. This may be because the carbon content affects the transformation of the crystalline phase of TiO_2_ by prohibiting the formation of the rutile phase.^34^ On the other hand, the absence of an N-CQDs peak in the XRD diffractogram of the N-CQDs/TiO_2_ nanocomposite can be explained by the very low amount of N-CQDs, the very good N-CQD dispersion in the TiO_2_ matrix, or less crystallization compared to TiO_2_.^35^ Using the Debye–Scherrer equation, the crystal size of TiO_2_ from the (101) diffraction plane of anatase TiO_2_ was found to be 15.16 nm for pure TiO_2_ and 10.38 nm for N-CQDs/TiO_2_ nanocomposites. Additionally, a lattice fringe with a *d*-spacing of 0.36 nm calculated from the Debye–Scherrer equation corresponds to the (101) tetragonal anatase phase TiO_2_ plane.

**Figure 3:**
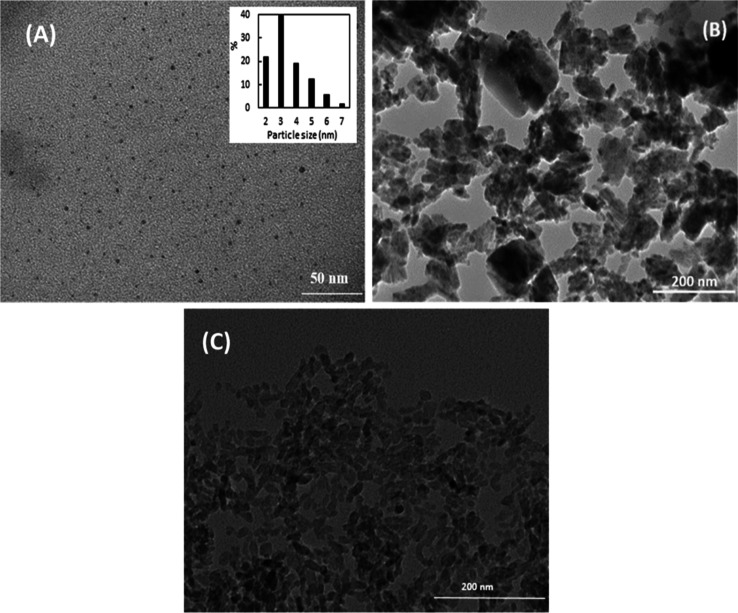
Representative TEM images of (a) N-CQDs, (b) bare TiO_2_, and (c) N-CQDs/TiO_2_.

Figure 3 shows representative TEM images of as-prepared N-CQDs and N-CQDs/TiO_2_ nanocomposites. From the TEM image of N-CQDs (Figure 3a), it can be concluded that many spherical-shaped nanoparticles with an average diameter of 3 nm and a nearly uniform size distribution were formed from the *R. crispus* L. plant by the presented method. It is noteworthy that the crystal size distribution is very close to the TEM image obtained for TiO_2_ (Figure 3b). The TEM micrograph of N-CQDs/TiO_2_ nanocomposites (Figure 3c) demonstrated that N-CQDs are well dispersed on the surface of TiO_2_, with an average particle size of 10 nm.

The surface morphologies of N-CQDs, TiO_2_, and N-CQDs/TiO_2_ samples were examined using SEM analysis. When the SEM images recorded for bare N-CQDs (Figure S1a,b) are examined, it can be concluded that agglomerated carbon crystal structures exist in separate phases. It can be concluded from Figure S1c,d that pure TiO_2_ nanoparticles, spherical in shape and approximately 10 nm in size, preserve their shape after the addition of N-CQDs but decrease in size. In Figure S1d, the presence of N-CQDs dispersed on the TiO_2_ crystal particles formed with a size of about 10 nm can be seen. On the other hand, from the EDS spectrum, it is understood that the N-CQDs/TiO_2_ nanocomposite is mainly composed of C, O, Ti, and N elements and is highly pure, and the C ratio is low compared to other elements. The C, O, Ti, and N contents of N-CQDs/TiO_2_ nanocomposite are 4.02, 51.84, 42.97, and 0.05%, respectively, while the O and Ti contents in TiO_2_ are 40.24 and 59.76%, respectively. The decrease in the Ti content in the N-CQDs/TiO_2_ nanocomposite structure reveals that the Ti atoms in TiO_2_ are successfully replaced by N-CQDs.

**Figure 4:**
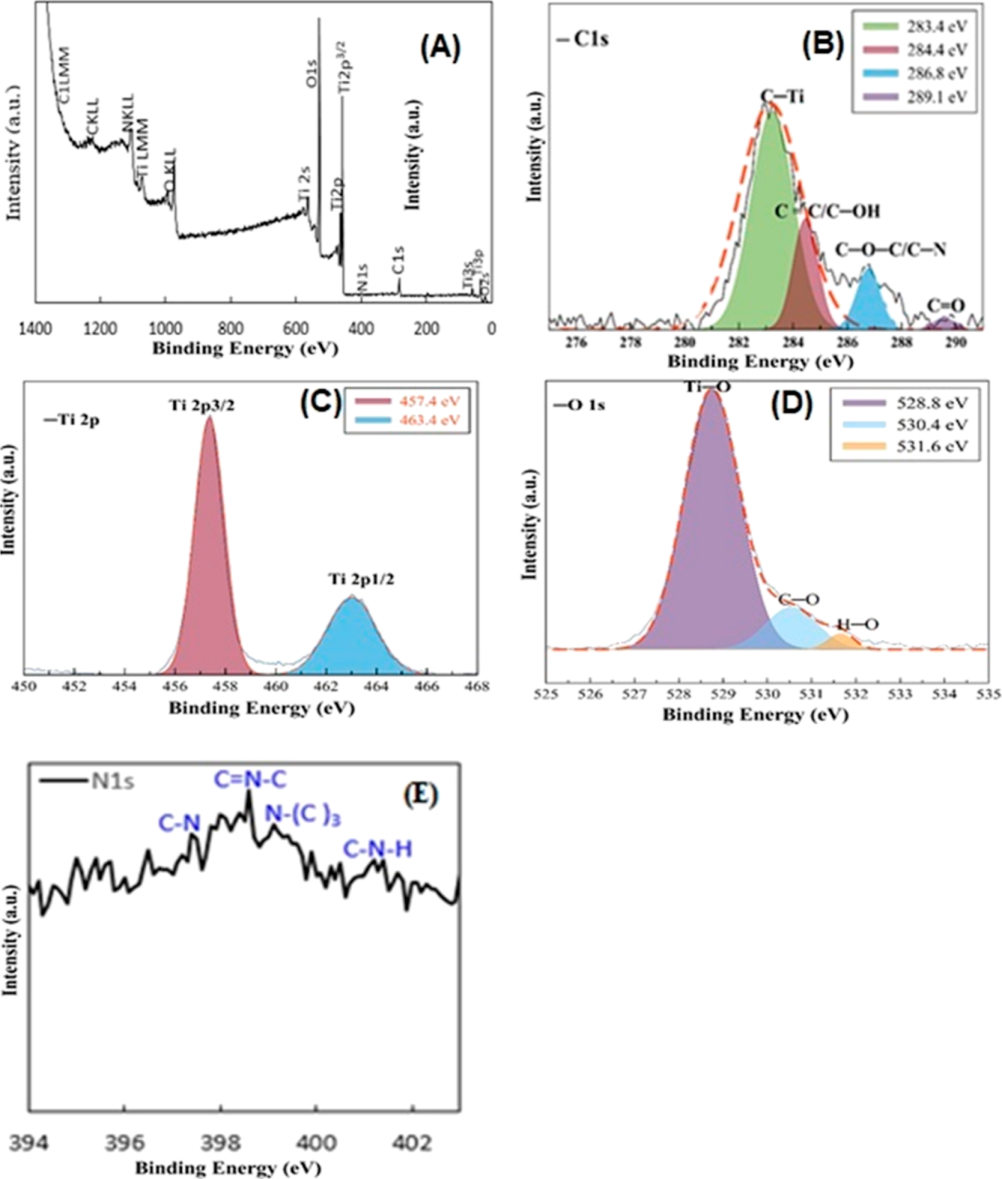
XPS survey spectra of as-prepared N-CQDs/TiO_2_ nanocomposites (a) and high resolution XPS spectra for C 1s (b), Ti 2p (c), O 1s (d), and N 1s (e).

XPS analysis was conducted on as-synthesized materials to reveal the surface chemical composition of N-CQDs, as shown in Figure S2. C 1s, N 1s, and O 1s atoms associated with 0D material were detected in the XPS survey spectrum, showing all expected elements existed in the N-CQDs structure (Figure S2a). In the high-resolution C 1s XPS spectrum, three main peaks were assigned as C=O/C=N, C–O/C–N, and C=C at binding energies of 288.5, 286.0, and 284.5 eV,^36^ respectively (Figure S2b). In parallel to this, the peaks at 531.8 (C=O) and 533.1 (C–O) eV were observed in the deconvoluted O 1s XPS spectrum^36^ (Figure S2c). XPS spectra of N-CQDs/TiO_2_ nanocomposites are given in Figure 4. It is understood from the XPS survey spectrum (Figure 4a) that N-CQDs/TiO_2_ nanocomposite consists of C (17.65%), N (1.86%), O (59.21%), and Ti (21.27%). The high-resolution XPS spectrum of the C 1s region (Figure 4b) mainly includes four typical asymmetrical peaks at binding energies of 283.4, 284.4, 286.8, and 289 eV, which are attributed to C–Ti, C–C (graphite)/C–OH, C–O–C/C–N, and O=C–O bonds^25^ respectively, implying the existence of the N-CQDs in the N-CQDs/TiO_2_ nanocomposites, which is well coherent with the HRTEM and FTIR results (vide infra). From these results, it is understood that carbon replaces some lattice titanium atoms and forms a Ti–O–C structure.^37^ The Ti 2p peak (Figure 4c) is deconvoluted into two peaks at binding energies of 457.4 eV (Ti 2p_3/2_) and 463.4 eV (Ti 2p_1/2_), corresponding to the Ti^4+^ 2p_3/2_ and Ti^4+^ 2p_1/2_ core levels. Because the difference between two lines is 0.7 eV, which is coherent with the +4 oxidation state.^25,38^ In the deconvoluted O 1s spectrum (Figure 4d), the peaks at 528.8, 530.4, and 531.6 eV were ascribed to the presence of Ti–O, C–O, and H–O bonds, respectively.^13^ Besides, the typical peaks located at 397.4, 398, 399.1, and 401.2 eV in the deconvoluted N1s spectrum (Figure 4e) of the N-CQDs/TiO_2_ nanocomposite are attributed to the C–N, C=N–C, N-(C)_3_, and C–N–H, respectively.^39,40^

FTIR analysis was performed on pristine N-CQDs, pristine TiO_2_, and N-CQDs/TiO_2_ nanocomposites to evaluate their surface functional groups. In the FTIR spectrum for N-CQDs (Figure S3), the broad peak between 3627 and 3300 cm^–1^ is assigned to both −OH and −NH stretching vibrations. The absorption bands at 598, 1101, 1170, 1390, 1506, 1631, 1702, 2806, 3002, and 3357 cm^–1^ are assigned to S–C bending vibration, C–O stretching, C–N bending, C–H bending, C–C bending, C=C stretching, COOH stretching, C–H_2_ symmetric stretching, −C–H_2_ asymmetric, and N–H stretching vibrations, respectively. These results explain the high-water solubility of N-CQDs containing numerous amine, hydroxyl, and carboxylic functional groups and their high ability to react with other components.^41−43^ It can be said that the peaks between 400 and 600 cm^–1^ are assigned by the Ti–O stretching band vibrations of TiO_2_.^13^ The shifting of the peak observed at 584.37 cm^–1^ in the FT-IR spectra of TiO_2_, resulting from the Ti–O vibration, to 611.37 cm^–1^ in N-CQDs/TiO_2_ clearly reveals that carbonaceous groups were introduced on the surface of TiO_2_, which confirmed the formation of the N-CQDs/TiO_2_ nanocomposites.

**Figure 5:**
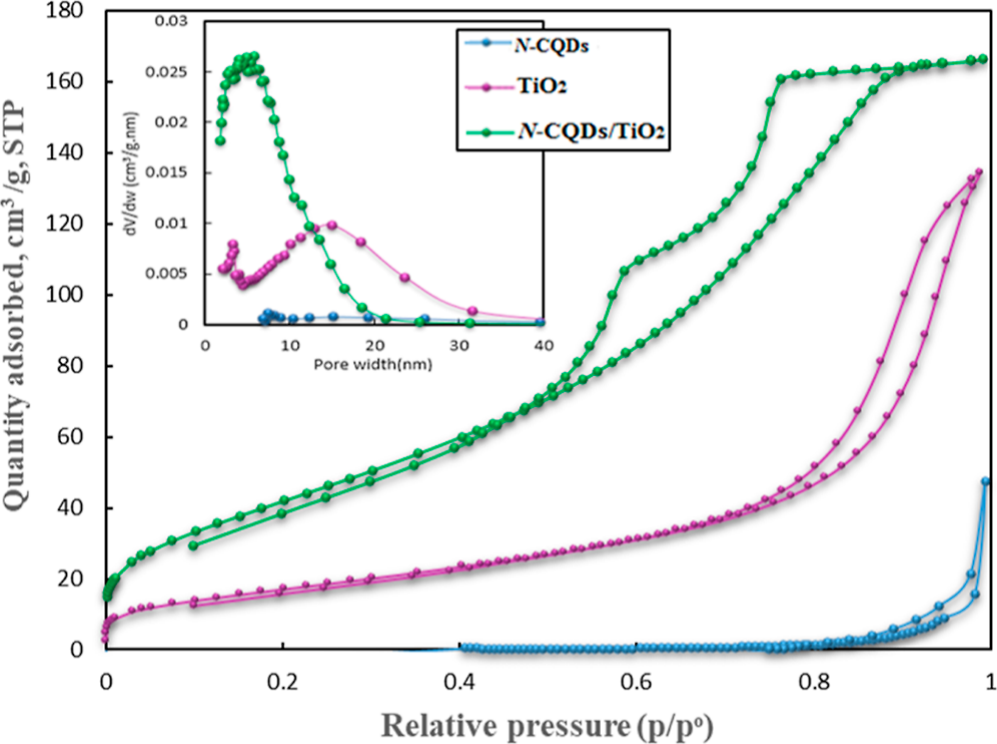
N_2_ adsorption–desorption isotherms for N-CQDs, TiO_2_, and N-CQDs/TiO_2_ nanocomposites. Inset: BJH pore size distribution of the corresponding samples.

Figure 5 exhibits N_2_ adsorption–desorption isotherms and BJH pore size distributions of N-CQDs, TiO_2_, and N-CQDs/TiO_2_ nanocomposites and their related BJH pore size distribution curves (the inset of Figure 5). Table S2 indicates the textural properties of the synthesized samples. Upon the analysis of Table S2 and Figure 5, it can be concluded that the as-synthesized samples exhibited a mesoporous character. For example, N-CQDs/TiO_2_ nanocomposites show a characteristic type IV isotherm with a pronounced H2-type hysteresis loop (Figure 5), which reflects the pore structure having an ink bottle-shaped mesoporous material with a relatively wide hole neck.^42^ As displayed in Figure 5, the isotherms of TiO_2_ and N-CQDs belong to the characteristic type IV isotherm with a hysteresis loop (H3-type) in the higher relative pressure range, which indicates the existence of numerous slit-like mesopores.^44^ The isotherm curve for N-CQDs illustrated a very minor hysteresis loop, a sign of some mesopores, possibly due to the aggregation of particles.^45^ The shift of the inflection point of *P*/*P*_o_ for N-CQDs/TiO_2_ compared to N-CQDs to lower relative pressures clearly indicates that the pore size is reduced as a result of the combination of N-CQDs and TiO_2_,^46^ as confirmed by Table S2 and Figure 5. The BET surface areas of N-CQDs, TiO_2_, and N-CQDs/TiO_2_ nanocomposites were specified as 0.18, 64.23, and 161.12 m^2^ g^–1^, and their corresponding pore volumes were 0.073, 0.209, and 0.262 cm^3^ g^–1^, respectively. In contrast, the average pore diameter of the N-CQDs/TiO_2_ (5 nm) sample was much smaller compared to the TiO_2_ (11.61 nm) and N-CQDs (38.79 nm). The greater surface area and larger pore volume of the N-CQDs/TiO_2_ nanocomposite are indications that the photocatalyst has more active centers, which provides it with the advantage of high photocatalytic efficiency. Adsorption is the first step of heterogeneous oxidation reactions and is followed by photocatalytic TC degradation, which produces reactive oxidative species via light irradiation.^47^

**Figure 6:**
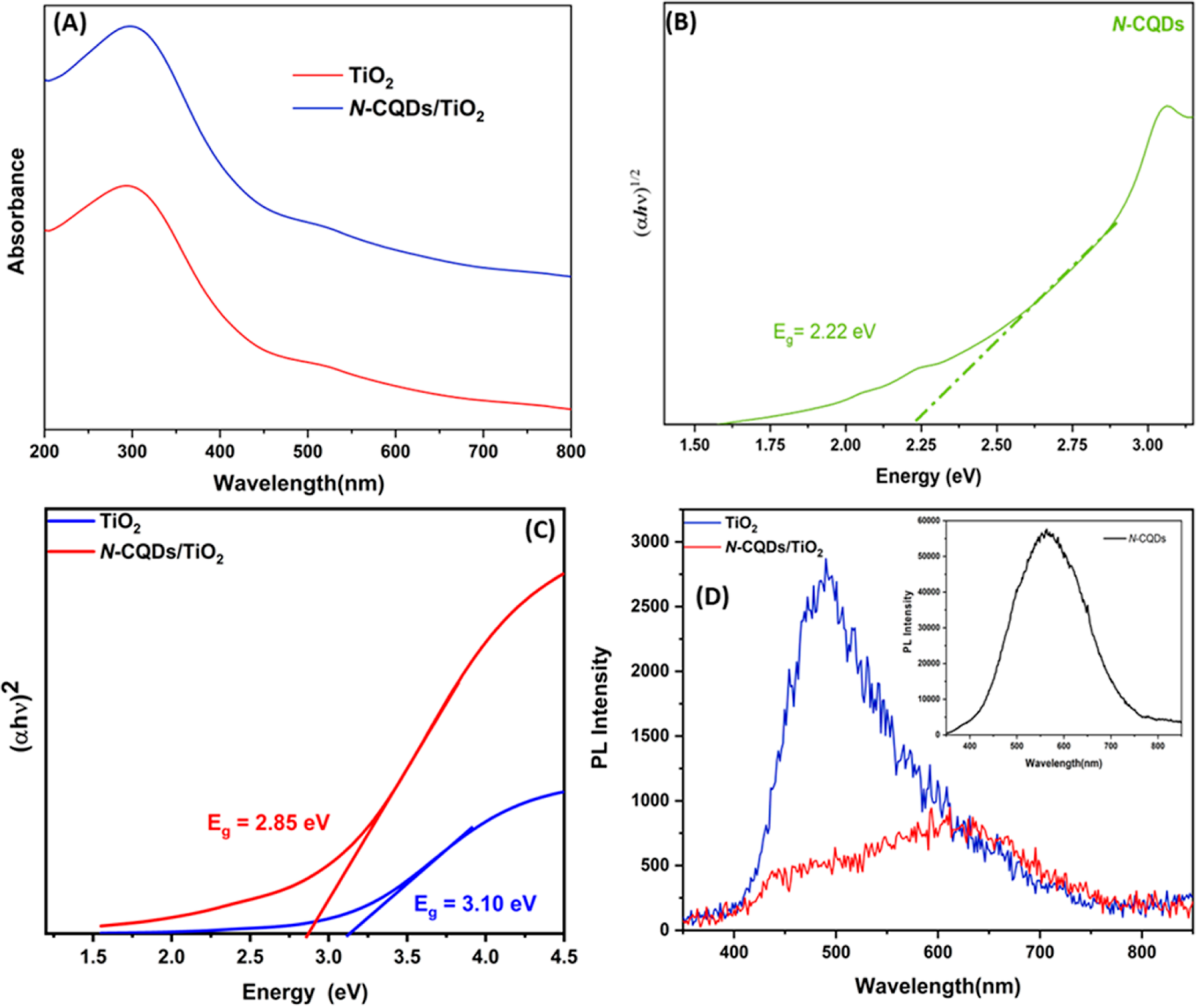
(a) UV–vis DRS spectra, (b,c) Tauc Plots of N-CQDs, TiO_2_, and N-CQDs/TiO_2_, and (d) PL spectra of the prepared samples.

UV–vis–NIR diffuse reflectance spectroscopy was used to evaluate the optical properties and calculate the optical bandgap values of TiO_2_ and N-CQDs/TiO_2_ samples. It can be seen from Figure 6a that while TiO_2_ gives an absorption band around 400 nm following its intrinsic bandgap absorption characteristic, the absorption edge shifts to 435 nm when TiO_2_ combines with N-CQDs. The light absorption shifting to the visible region can be attributed to the chemical interactions of Ti–O–C bonds between TiO_2_ and N-CQDs.^48^ From Figure 6a, it can be seen that the absorption intensity of TiO_2_ is high in the ultraviolet region, but after doping with N-CQDs, the absorption band becomes redshifted, and absorption occurs in both the ultraviolet and visible regions with a much greater intensity than that of TiO_2_. It can be deduced from these results that the increase in light absorption is due to photosensitivity due to the N-CQDs.^49,50^

Band gap energy (*E*_g_) of pristine TiO_2_ and N-CQDs/TiO_2_ nanocomposites was predicted by eq 1.^51^
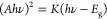
1where *A*, *K*, *h*ν, and *E*_g_ refer to the absorption coefficient, a constant, the energy of a photon (eV), and the band gap, respectively. *E*_g_ of the samples was estimated by extrapolating the linear part of the plot of (α*h*ν)^2^ values versus hv (Figure 6b,c). These values were found to be 2.22, 3.10, and 2.85 eV for N-CQDs, pristine TiO_2_, and N-CQDs/TiO_2_ nanocomposite, respectively. Accordingly, pristine TiO_2_ can only absorb light in the UV region, while it shows more absorption in both the UV and visible regions after being modified with N-CQDs. This may be a reason for the high photocatalytic efficiency of the N-CQDs/TiO_2_ photocatalyst, which is consistent with the results publicized by Wang et al.^50^ They examined the photocatalytic oxidation of RhB both under UV and visible light irradiation with the TiO_2_-CQD catalyst. They reported that almost all of the RhB was removed under UV radiation, while only 76% was removed under visible light irradiation under the same conditions and time.

On the other hand, photoluminescence spectroscopy (PL) is an efficient technique to get insights into the behavior of photogenerated charge carriers (e^–^/h^+^) and their recombination possibilities. Because PL emission is usually caused by signals initiated by the recombination of the electron–hole pairs. It is estimated that the lower the emission intensity, the lower the probability of recombination of electron–hole pairs and thus the higher the photocatalytic efficiency of the catalyst.^13^Figure 6d exhibits the room temperature PL spectra for the samples of N-CQDs, pure TiO_2_, and N-CQDs/TiO_2_ nanocomposites with an excitation wavelength of 325 nm. Remarkably powerful PL emission spectra (inset) were monitored at 563 nm for TiO_2_, while that in the case of N-CQDs/TiO_2_ was lowest due to the existence of the more delocalized electrons in N-CQDs. Moreover, the lower-energy redshift of the strongest peak of fluorescence recombination for N-CQDs/TiO_2_ nanocomposite reveals that the bandgap energy of TiO_2_ nanoparticles doped with N-CQDs particles is lowered. The results were consistent with the photocatalytic activities of the prepared materials. The higher photocatalytic activity of the N-CQDs/TiO_2_ nanocomposite revealed that the coupling of N-CQDs with TiO_2_ encouraged the separation of photo-generated charge transporters by electron transfer reactions, thereby enhancing the photocatalytic efficiency of the N-CQDs/TiO_2_ photocatalysts. This result is attributed to the proficient electron transport in the interfacial region between N-CQDs and TiO_2_, thus inhibiting carrier recombination.^43,50^

### 3.2 Contribution of Various Processes to TC Removal Efficiency

**Figure 7:**
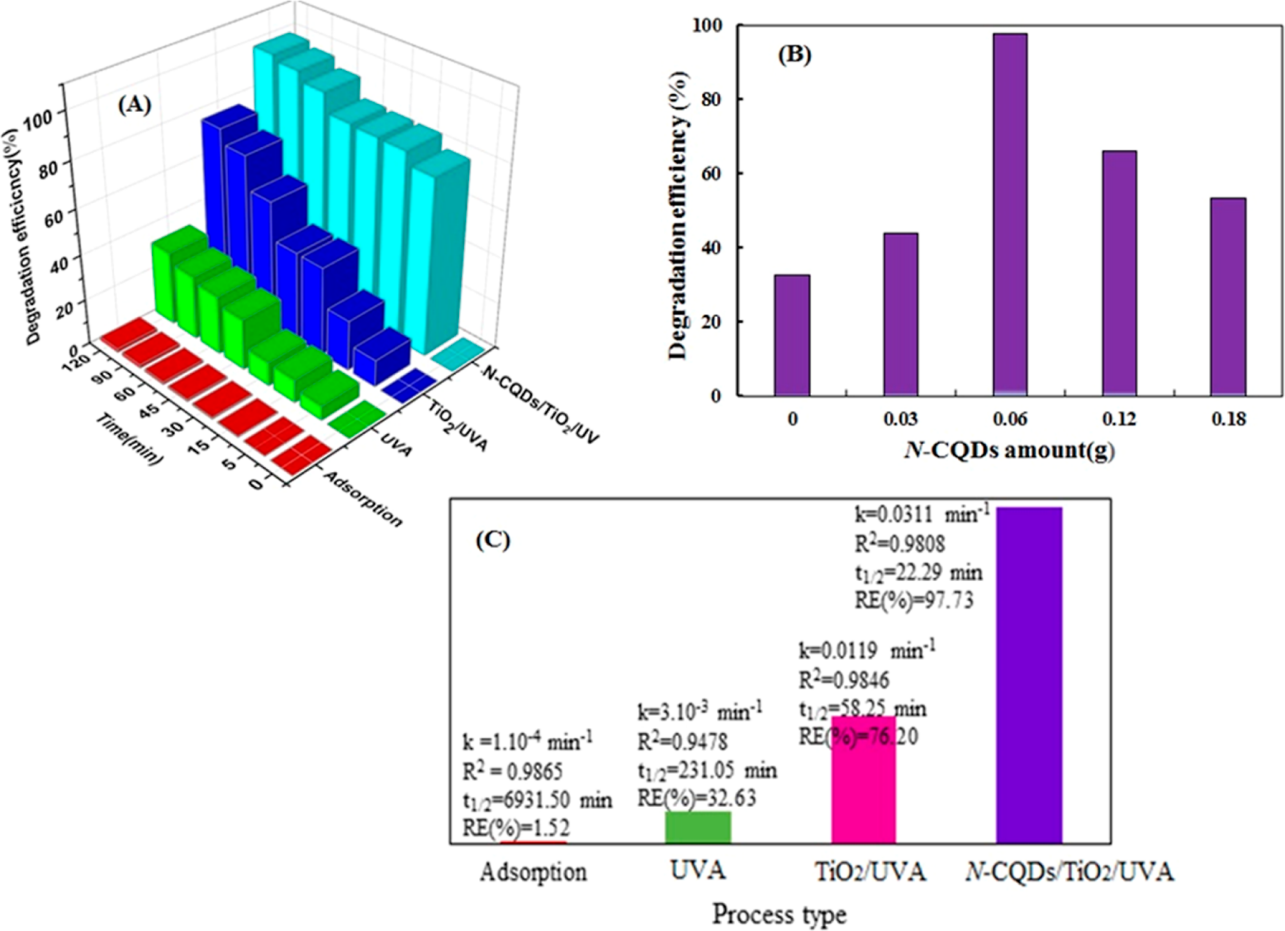
(a) Effect of different processes on TC degradation. (b) Effect of N-CQD amount on the photocatalytic activity of prepared N-CQDs/TiO_2_ catalysts for TC removal. (c) Removal efficiencies and kinetic parameters for TC removal via different processes. Conditions: [catalyst]_0_ = 0.2 g/L, [TC]_0_ = 10 mg/L, and pH = 5.15.

The effect of different parameters on the TC removal efficiency with different N-CQD contents was evaluated under UVA irradiation and at the optimized conditions previously determined as 10 mg/L TC, 0.2 g/L N-CQDs/TiO_2_,120 min, and a pH of 5.15 (natural pH), and the results are illustrated in Figure 7a. According to the results, the adsorption of TC molecules on the N-CQDs/TiO_2_ surface was only 1.52% over 120 min, implying that the contribution of adsorption in percent TC removal was not important. Depending on Figure 7a, it can be said that the TC removal under UVA light alone (photolysis) (32.63%) is not effective enough to remove the target pollutant acceptably. Whereas UVA irradiation in the presence of TiO_2_ nanoparticles resulted in significant removal of TC (76.2%) compared to the use of the photolysis process alone. If N-CQDs/TiO_2_ is used as a catalyst, TC removal of 97.73% in the same period reveals that N-CQD nanoparticles have a positive effect on increasing the photocatalytic activity of TiO_2_. This excellent removal efficiency can be attributed to the unique photon conversion and charge transport characteristics of N-CQDs nanoparticles.^50,52^ The improvement in the photocatalytic activity of TiO_2_ upon its combination with N-CQDs can be attributed to the following reasons. First, due to the very good electron acceptor and carrier properties of N-CQDs that prevent/reduce e/h^+^ recombination, which will give the e/h^+^ pairs enough time to generate more ROS species having an active role in the oxidation of pollutants. Second, N-CQDs absorb light in both UV and visible regions very well and convert the long wavelength light they absorb into shorter-wavelength light with higher energy. These high-energy photons will activate TiO_2_ more effectively. Therefore, it will help to increase the photoactivity of the TiO_2_ photocatalyst.^50^

Thereafter, the impact of N-CQD amount on the photocatalytic efficiency of the prepared N-CQDs/TiO_2_ catalyst for TC removal was studied under UVA irradiation. The amount of N-CQDs added was changed to 0, 0.03, 0.06, 0.12, and 0.18 g while keeping the amount of TiO_2_ in the catalyst constant (Figure 7b). As shown, by enhancing the amount of N-CQDs from 0.03 to 0.06 g, the percent TC degradation rose from 43.85 to 97.73%, probably due to the high light harvesting and improved separation efficiency of photoproduced electron/hole pairs. When the amount of N-CQDs was raised from 0.06 to 0.12 g and 0.18 g, the removal efficiency dropped from 97.73 to 66.25% and 53.48%, respectively. The reason for the decrease in photocatalytic efficiency is (a) a decrease in the amount of light absorbed by the composite due to competition for light absorption between the increasing particles of N-CQDs and TiO_2_, (b) excessive N-CQDs particles can reduce photocatalytic efficiency by taking part in the recombination of photogenerated e^–^/h^+^ pairs, and (c) it can be said that the N-CQDs nanoparticles accumulated on the surface and pores of TiO_2_ create an obstacle in the transfer of the solution to the surface.^52^ Therefore, 0.06 g was maintained as the optimum N-CQD amount in all the surviving steps of the study.

We wondered what the activity of the catalyst would be by using another natural source instead of *R. crispus* L. as a natural herbal source, and for this purpose, we conducted experiments for TC removal with N-CQDs/TiO_2_ nanocatalyst prepared from a potato with the same procedure under the same optimum conditions. As a result of the experiments, we have seen that there is 85% TC removal under optimum conditions with the N-CQDs/TiO_2_ catalyst prepared from potatoes, which is lower than the efficiency of the N-CQDs/TiO_2_ catalyst from *R. crispus* L. (97.73%). It can be said that the reason for this is that potato and *R. crispus* L. plants have different carbon atom numbers, carbon chain lengths, and functional groups, which play an important role in the hydrothermal carbonization process and hence in the formation of N-CQD nanoparticles with different properties.^15^ Based on this result, it is understood that N-CQDs prepared from *R. crispus* L. are more active in TC removal by photocatalytic process than N-CQDs prepared from potato after combination with TiO_2_.

The reaction kinetics of TC removal via different processes were examined by applying a linear pseudo-first-order model (eq 2).
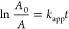
2
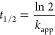
3where *A*_0_ and *A*_t_ are the absorbance values of residual TC at time zero and time *t*, respectively, *k*_app_ is the apparent reaction rate constant, and *t* is the reaction time for the experiment.^34^Figure 7c illustrates the referring kinetic parameters (apparent rate constant, *k*_app_, half-life, *t*_1/2_, and linear regression coefficients, *R*^2^) for different processes. Figure 7c and all linear regression coefficients (*R*^2^) demonstrate that results obtained from all of the process experiments fit into the pseudo-first-order kinetics.

The values of the calculated *t*_1/2_ from eq 3 for N-CQDs/TiO_2_ (adsorption), UVA (photolysis), TiO_2_/UVA, and N-CQDs/TiO_2_/UVA systems are 6931.50, 231.05, 58.25, and 22.29 min, respectively. As denoted in previous studies, the shorter the half-time and the higher the apparent rate constant, the faster the charge transfer and the fewer electron/hole recombinations. The process with the highest *k*_app_ and the lowest *t*_1/2_ value is regarded as the most efficient. In this study, in parallel with the increase in TC removal efficiency, *k*_app_ value increased, while *t*_1/2_ value decreased according to the order of adsorption, UVA, TiO_2_/UVA, and N-CQDs/TiO_2_/UVA processes.^34^ From Figure 7c, it is understood that the photocatalytic process, in which N-CQDs/TiO_2_ is used as a catalyst, is the most effective process with a rate constant of 0.0311 min^–1^ and a half-life of 22.29 min.

### 3.3 Impact of Experimental Parameters on the Photocatalytic Oxidation of Tetracycline in the Presence of N-CQDs/TiO_2_ Nanocomposites

The effect of some variables on the photocatalytic degradation of TC is discussed below, and a possible mechanism for the degradation of TC by the N-CQDs/TiO_2_ photocatalyst is proposed.

#### 3.3.1 Impact of N-CQDs/TiO_2_ Dosage

Determining the optimum catalyst concentration is an important parameter in practical applications to ensure effective photon absorption and reduce light scattering.^47,52^Figure S4a represents the impact of catalyst amount on the percent TC degradation. The experiments were carried out at concentrations ranging from 0.05 to 0.6 g/L while protecting all other operational parameters constant (10 mg/L TC and native pH). As the catalyst concentration was incremented from 0.05 to 0.2 g/L, percent TC degradation was also found to be gradually enhancing from 59.35 to 97.73% in 120 min due to the more available active sites for adsorption on the surface. However, the further increase in catalyst amount caused a reduction in the percentage degradation of TC. Because the higher catalyst loading causes more light scattering, which diminishes the photoproduction output of e^–^/h^+^ pairs.^47^

#### 3.3.2 Impact of TC Concentration

As displayed in Figure S4b, the photocatalytic performance of the N-CQDs/TiO_2_ nanocomposite at TC degradation was appreciably affected by the initial TC concentration. When the TC concentration was raised from 5 to 30 mg/L, the corresponding percentage degradation was prominently reduced from 98.83 to 64.12%. As expected, the chance of a reaction between TC molecules and reactive species should increase as the initial TC concentration rises. However, it should be taken into account that the reactive species produced by the fixed amount of catalyst will not be sufficient for the increased TC molecules and that there will be more TC, intermediates, and photogenerated species competing to be adsorbed on the surface area of the fixed amount of catalyst. In this case, TC removal decreases significantly with increasing TC concentration.^47,52^

#### 3.3.3 Impact of Acidity of Medium on TC Degradation

As shown in Figure S4c, the percentage of degradation of TC by the N-CQDs/TiO_2_ nanocomposite varies with changing initial solution pH. Because pH affects both the surface charge of photocatalysts, the degree of ionization of TC, and the production of reactive oxygen species, it has a remarkable impact on photocatalytic oxidation.^5^ To analyze the percent TC degradation at the various pH values in the aqueous solutions, experiments which examine the TC degradation and zeta potential with different pH were designed. As displayed in Figure S4c, the lowest degradation efficiency was at a solution pH of 2 (8.59%). The TC degradation efficiency, which was 78.09% at pH 3, reached 97.73, 89.19, 92.08, and 97.91% when the initial solution pH was raised to 5.15 (natural pH), 7, 9, and 11, respectively.

The zero point of charge (pH_zpc_) of N-CQDs/TiO_2_ nanocomposite is 6.6 (see Figure S4d). That is to say, the surface of the photocatalyst will remain positively charged below this pH, whereas it will be negatively charged at a pH higher than 6.6. Additionally, TC is an amphiprotic molecule with dissociation constants of 3.32, 7.78, and 9.7, which have a different form in each of them.^5^ Since both the surface of the catalyst and the TC molecules are positively charged at pH 2, the repulsive force between them prevents adsorption. In addition, Cl^–^ from HCl used in pH adjustment inhibits degradation by having a scavenger effect. The same is true for pH 3. However, although the surface charges of the photocatalyst and TC molecules did not change, the degradation efficiency reached 78.09% as the solution pH rose from 2 to 3, revealing that an adsorption took place between the acidic phenol, enol, and amine groups of the TC molecules and the photocatalyst molecules via hydrogen bonds. The removal efficiency, which enhanced to 97.73% at pH 5.15, can be attributed to the electrostatic interaction and hydrogen bonding between the photocatalyst surface and the TC molecules in the form of TCH_2_^±^. The small reduction in TC degradation at pH 7(89.19%) indicates some decrease in electrostatic interaction between the negative charge of the catalyst surface and TC molecules. As the pH rose to 9 and 11, the removal efficiency enhanced again and reached the values of 92.08 and 97.91%. Although repulsion forces dominate between the negatively charged photocatalyst and negatively charged TCH^–^ and TC^2–^ molecules, the increase in removal efficiency can be explained as follows. The acidity of the environment also affects the number of hydroxyl radicals (OH•) formed as a result of the reaction between h^+^ and H_2_O/OH^–^. While the amount of OH^•^ radicals is higher in the reaction at basic pH values, the h^+^ type is dominant in the reactions at acidic and neutral pH values.^47^ Accordingly, the high removal efficiency at pH 9 and pH 11 may be due to the high amounts of OH^•^ radicals at these pH values. Considering all the abovementioned results, all of the other ongoing experiments in this study were performed at a pH value of 5.15, which is the natural pH of TC solution. Moreover, not using acid or base to adjust pH in experiments is important in terms of preventing side effects.

#### 3.3.4 Band Alignments

Before proposing a reasonable mechanism for the photodegradation of TC, defining the band edges of components in nanocomposite is one of the vital aspects that will help. To achieve this, the band alignments of bare materials were calculated with the help of valence band (VB)-XPS analysis and the Tauc plots methods. The value associated with the intersection of the tangent and oblique lines close to the 0 point of the *X*-axis is known as the valence band potential measured by VB-XPS (Figure S5a,b).^53^ When the intersection of related results was drawn, 2.45 and 1.88 eV values were found for N-CQDs and TiO_2_, respectively, and were converted to standard hydrogen electrode potential (*E*_VB-NHE_) following the well-known eq 4
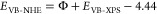
4where *E*_NHE_, Φ, and *E*_VB-XPS_ can be defined as the standard electrode potential, electron working function of the XPS analyzer (4.543 for the device in our case), and the VB value obtained with VB-XPS, respectively.^53^ The VB values for N-CQDs and TiO_2_ were determined using this formula to be 2.55 and 1.99 V, respectively. Combining VB-XPS and Tauc plot results with the well-known equation (*E*_g_ = *E*_VB_ – *E*_CB_),^54^ the CBs of N-CQDs and TiO_2_ were found to have 0.38 and −1.11 V, respectively, and were represented schematically in Figure S5c.

**Figure 8:**
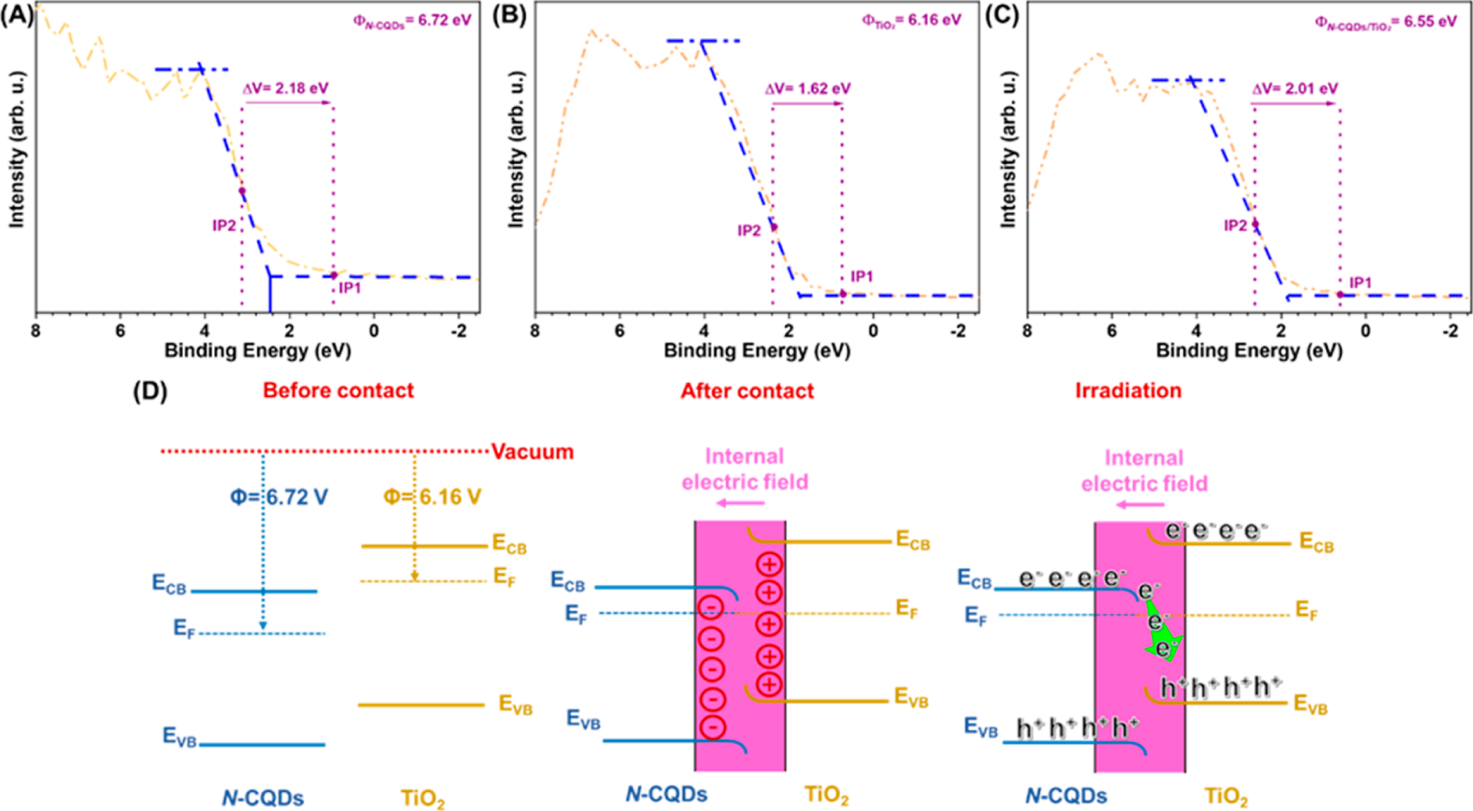
Work functions of (a) N-CQDs, (b) TiO_2_, (c) N-CQDs/TiO_2_, and (d) IEF between semiconductors before and after contact and under irradiation resulted in bending of band edges for N-CQDs and TiO_2_.

Their charge transfer direction at the component interfaces is yet another critical criterion in addition to their band alignments. The function of the associated band edges may be understood experimentally by analyzing the work functions of N-CQDs and TiO_2_. The work function values for N-CQDs, TiO_2_, and N-CQDs/TiO_2_ were determined using VB-XPS, as shown in Figure 8a–c, respectively. Where materials encounter one another at their interfaces, the magnitude of a work function controls the direction of charge flow. It is simpler to accept an electron from a material with a small work function because the bigger the work function, the farther the Fermi level is from the vacuum level of the corresponding material.^2,55^ The electron-donor material’s surface becomes positively charged, and vice versa, throughout this charge transfer until their Fermi levels (*E*_F_) are in balance. The associated work function of each component is determined using the equation (Δ*V* = Φ – φ) (Φ is the work function of the material, and φ is 4.543 eV for the instrument).^56^ From the relationship through the inflection point (IP1; change in binding energy at the baseline; and IP2; the middle point of Fermi energy distribution) distance, Δ*V* can be calculated.^7,57^ By following this, 6.72, 6.16, and 6.55 eV values were obtained for N-CQDs, TiO_2_, and N-CQDs/TiO_2_, respectively (Figure 8a–c). When contact is established among the components, TiO_2_ with a smaller work function than that of N-CQDs is highly favorable to donate electrons; thereby, N-CQDs accept electrons until balancing *E*_F_. This opposite charge distribution at the interfaces generates an internal electric field (IEF)^58^ and the band edges of TiO_2_ can be assumed to bend upward, while the band edges of N-CQDs can bend downward (Figure 8d). As a whole, related band edges (between CBs and VBs of N-CQDs and TiO_2_) for the required charge flow resulted in mismatched spatial bending. The recombination of photogenerated electrons on the CB of N-CQDs and photogenerated holes on the VB of TiO_2_ would just be promoted throughout the UV-A irradiation, caused by IEF and the band bending. In this situation, while the electrons and holes at the two ends stay in their original energy bands, the holes in the VB of TiO_2_ and the electrons in the CB of N-CQDs may readily migrate to the interface for the coupling of materials. This results in the formation of a typical S-scheme heterojunction that makes charge separation and transfer easier.^2,59^ It is obvious that the charge carrier redistribution process revolves around the heterogeneous interface. The total charge migration path of the nanocomposite can be proposed as an S-scheme mechanism,^60^ and the superior photocatalytic activity of N-CQDs/TiO_2_ in addition to their high photogenerated charge separation features are consistent with the results.

#### 3.3.5 Influence of Scavengers and Photodegradation Mechanism

To identify the capacity of alternative active species in the photocatalytic degradation of TC and to explore the reaction mechanism, we enforced radical capturing experiments under the same reaction procedure by adding various trap compounds to the N-CQDs/TiO_2_/aqueous TC solution system. [TC]/[trap] ratio was 1:1. KI, Na_2_SO_4_, EDTA-Na_2_, isopropyl alcohol (IPA), and benzoquinone (ρ-BQ) are served as the scavengers of free and surface OH^•^ radical, h^+^, and OH^•^ radical, h^+^, free OH^•^ radical and h^+^, O_2_^•–^ (superoxide) radical, respectively,^6,17,61−63^ and the results are illustrated in Figure 4e. As seen in Figure 4e, at the irradiation time of 120 min, the percent TC degradation was reduced from 97.73 to 81.83, 76.85, 72.81, 68.93, and 58.36% in the presence of KI, IPA, Na_2_SO_4_, EDTA, and BQ, respectively. In comparison to TC degradation without scavenger, as seen in Figure S4e, the introduction of KI slightly depressed the photocatalytic efficiency of N-CQDs/TiO_2_ nanocatalyst, indicating that OH_ads_^•^ and OH_free_^•^ radicals in solution have a slight influence on the degradation performance of the N-CQDs/TiO_2_ photocatalyst. The important decline in percentage degradation of TC after EDTA and BQ are introduced suggests that h^+^ and O_2_^•–^ radicals are the major effective species in the TC photooxidation process. The results obtained with the addition of IPA and Na_2_SO_4_ also support the conclusion that the role of OH^•^ radicals in photocatalytic degradation of TC is less. Accordingly, based on the preliminary results, it can be said that TC molecules are predominantly degraded by h^+^ and O_2_^•–^ radicals produced in N-CQDs/TiO_2_, followed by OH^•^ radicals.

**Figure 9:**
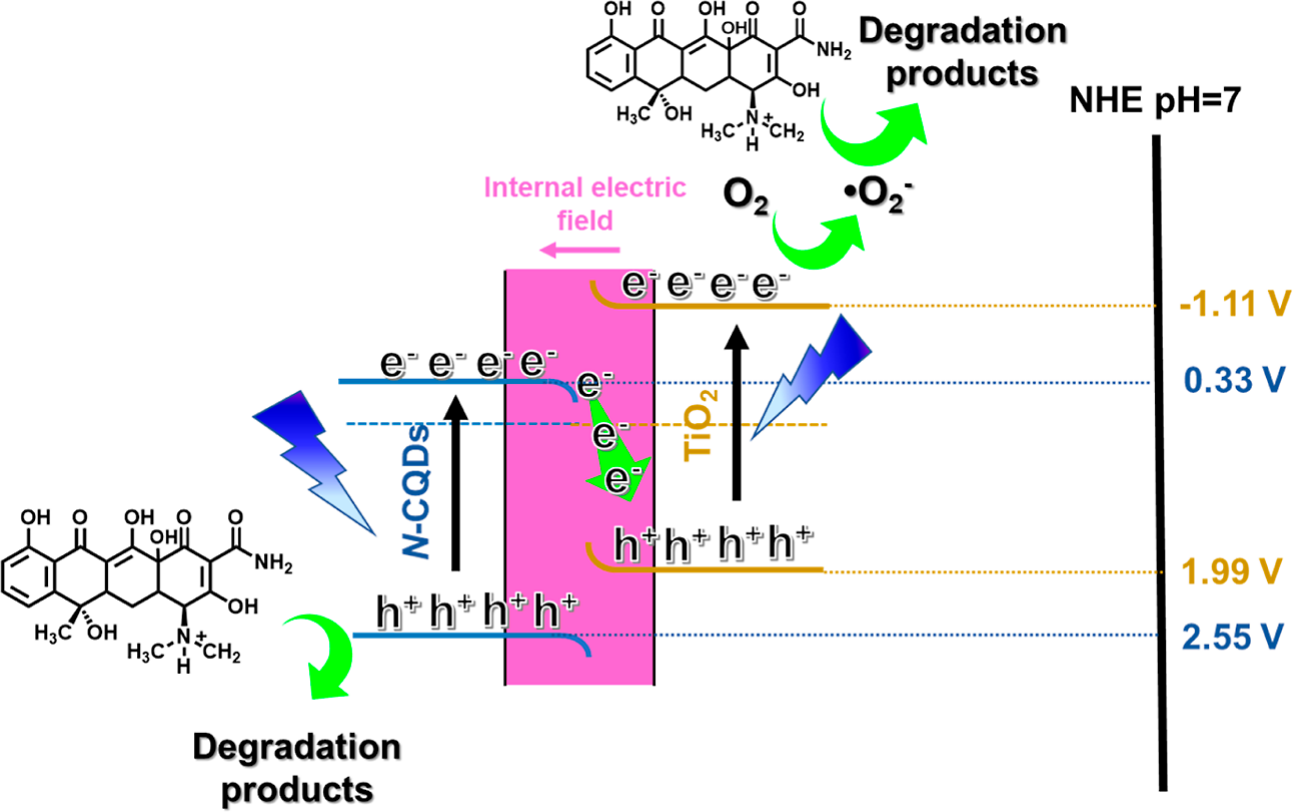
Schematic representation of the proposed mechanism in the N-CQDs/TiO_2_ nanocomposite for TC photodegradation.

On the basis of the above results related to scavenger experiments and band alignments, a plausible mechanism for the expedited charge separation and improved photocatalytic efficiency of the N-CQDs/TiO_2_ photocatalyst is exhibited in Figure 9. It can be seen from Figure 6a that TiO_2_ only absorbs light in the ultraviolet region, while the N-CQDs/TiO_2_ nanocomposite can absorb light in both the ultraviolet and visible regions due to the synergistic effect among the materials. In addition to photon harvesting ability, using the maximum band potential of the nanocomposite with an S-scheme mechanism not only causes high photogenerated charge separation but also overcomes the required thermodynamic redox transformation. As suggested in Figure 8d under irradiation, the photogenerated electrons of N-CQDs recombine with the photogenerated holes of TiO_2_; thus, the CB of N-CQDs is favorable for the photoreduction reaction, while the VB of TiO_2_ is for the photooxidation reaction.
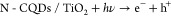
5

In this regard, after the generation of e^–^/h^+^ pairs (eq 5), the super oxide anion radical (O_2_^•–^) is formed via electron transfer to molecular oxygen (eq 6) in the CB of TiO_2_.
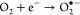
6

In parallel to this, holes remaining in the valence band of N-CQDs combine with H_2_O and turn into OH^•^ radicals (eq 7).
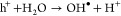
7

According to scavenger experiments with BQ, this reactive species degrades TC pollutants into small fragments (eq 8). TC molecules are decomposed either by the formed OH^•^ radicals or directly by h^+^ (eqs 6 and 10). According to the scavenger experiment results, h^+^ is mainly responsible for TC degradation.
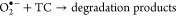
8
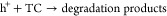
9
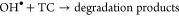
10

Building upon our prior studies^6,54^ on the photodegradation mechanism pathway analysis of TC, plausible mechanisms have been proposed to elucidate the photodegradation process. It has been confirmed that no intermediates with aromatic structures persist in the final products of TC photodegradation; instead, they undergo breakdown into minuscule, undetectable molecules such as H_2_O, CO_2_, NH_3_, and similar species. Finally, in order to demonstrate the superior performance of our catalyst in TC photocatalytic degradation, the results obtained from this study were compared with the results of other studies that previously performed the photocatalytic degradation of TC with different catalysts. Table S3 compares the photocatalytic TC degradation performance of the synthesized N-CQDs/TiO_2_ photocatalyst with the previously published studies. Comparing the performance of this photocatalyst to previous results revealed that this catalyst has remarkable performance in photocatalytic TC degradation.

## 4 Conclusions

In this study, plant-based N-CQDs were prepared and combined with TiO_2_ to yield N-CQDs/TiO_2_ heterojunction photocatalysts via a simple hydrothermal method. N-CQDs/TiO_2_ nanocomposites execute the efficient photocatalytic oxidation of TC in contaminated water under UVA light irradiation through the decoration of N-CQDs. As-synthesized N-CQDs, TiO_2_, and N-CQDs/TiO_2_ nanocatalysts were analyzed by various advanced analytical techniques, and the results disclosed successful doping of N-CQDs on the TiO_2_ surface. Compared to pure TiO_2_, the incorporation of N-CQDs onto the TiO_2_ surface significantly improved the visible light harvesting ability and photocatalytic performance by supplying 97.73% TC degradation in 120 min under the optimized conditions. Kinetic investigations on the TC degradation indicated that they follow the pseudo-first-order reaction. From the capturing experiments, it was understood that photo-produced O_2_^•–^, h^+^, and OH^•^ reactive oxidative species decompose TC into smaller intermediates and even H_2_O and CO_2_. The charge transfer direction at the interfaces of N-CQDs and TiO_2_ is a critical factor that affects the photocatalytic activity of the nanocomposite. The work function values of N-CQDs and TiO_2_ were analyzed to understand their band edges and their charge flow directions at the interfaces. It was proposed that an S-scheme heterojunction formation between N-CQDs and TiO_2_ leads to charge separation and transfer, which results in the superior photocatalytic activity of N-CQDs/TiO_2_. Depending on the obtained results, N-CQDs/TiO_2_ as an influential and green synthesized photocatalyst has great potential applications in the photocatalytic degradation of TC and other organic pollutants. The results can confirm the potential of N-CQDs/TiO_2_ photocatalysts in various photocatalytic applications, motivate further research in this field, and shed light on the synthesis of new photocatalysts with suitable band alignments for the removal of TC and similar organic compounds.

## Supporting Information Available

Additional data for materials characterization (SEM, XPS, FTIR, BET, and band alignment calculations (XPS-VB)), detailed procedures for the synthesis of materials, and effect of some parameters on photodegradation performance (PDF)

## References

[ref1] Bojer C.; Schöbel J.; Martin T.; Ertl M.; Schmalz H.; Breu J. Clinical Wastewater Treatment: Photochemical Removal of an Anionic Antibiotic (Ciprofloxacin) by Mesostructured High Aspect Ratio ZnO Nanotubes. Appl. Catal., B 2017, 204, 561–565. 10.1016/j.apcatb.2016.12.003.

[ref2] Wang L.; Liu Y.; Lin Y.; Zhang X.; Yu Y.; Zhang R. Z-scheme Cu2(OH)3F nanosheets-decorated 3D Bi2WO6 heterojunction with an intimate hetero-surface contact through a hydrogen bond for enhanced photoinduced charge separation and transfer. Chem. Eng. J. 2022, 427, 131704 10.1016/j.cej.2021.131704.

[ref3] Zhang Z.; Lan H.; Liu H.; Qu J. Removal of Tetracycline Antibiotics from Aqueous Solution by Amino-Fe (III) Functionalized SBA15. Colloids Surf., A 2015, 471, 133–138. 10.1016/j.colsurfa.2015.02.018.

[ref4] Zhao Y.; Gu X.; Gao S.; Geng J.; Wang X. Adsorption of Tetracycline (TC) onto Montmorillonite: Cations and Humic Acid Effects. Geoderma 2012, 183–184, 12–18. 10.1016/j.geoderma.2012.03.004.

[ref5] Yue L.; Wang S.; Shan G.; Wu W.; Qiang L.; Zhu L. Novel MWNTs–Bi2WO6 Composites with Enhanced Simulated Solar Photoactivity toward Adsorbed and Free Tetracycline in Water. Appl. Catal., B 2015, 176–177, 11–19. 10.1016/j.apcatb.2015.03.043.

[ref6] Kılıç D.; Sevim M.; Eroğlu Z.; Metin Ö.; Karaca S. Strontium Oxide Modified Mesoporous Graphitic Carbon Nitride/Titanium Dioxide Nanocomposites (SrO-Mpg-CN/TiO2) as Efficient Heterojunction Photocatalysts for the Degradation of Tetracycline in Water. Adv. Powder Technol. 2021, 32, 2743–2757. 10.1016/j.apt.2021.05.043.

[ref7] Shi H.; He Y.; Li Y.; He T.; Luo P. Efficient Degradation of Tetracycline in Real Water Systems by Metal-Free g-C3N4 Microsphere through Visible-Light Catalysis and PMS Activation Synergy. Sep. Purif. Technol. 2022, 280, 119864 10.1016/j.seppur.2021.119864.

[ref8] Zhang J.; Yuan X.; Jiang L.; Wu Z.; Chen X.; Wang H.; Wang H.; Zeng G. Highly Efficient Photocatalysis toward Tetracycline of Nitrogen Doped Carbon Quantum Dots Sensitized Bismuth Tungstate Based on Interfacial Charge Transfer. J. Colloid Interface Sci. 2018, 511, 296–306. 10.1016/j.jcis.2017.09.083. 29031149

[ref9] Huo P.; Ye Z.; Wang H.; Guan Q.; Yan Y. Thermo-Responsive PNIPAM@AgBr/CSs Composite Photocatalysts for Switchable Degradation of Tetracycline Antibiotics. J. Alloys Compd. 2017, 696, 701–710. 10.1016/j.jallcom.2016.11.219.

[ref10] Di J.; Xia J.; Ji M.; Xu L.; Yin S.; Chen Z.; Li H. Bidirectional Acceleration of Carrier Separation Spatially via N-CQDs/Atomically-Thin BiOI Nanosheets Nanojunctions for Manipulating Active Species in a Photocatalytic Process. J. Mater. Chem. A 2016, 4, 5051–5061. 10.1039/C6TA00284F.

[ref11] Bozetine H.; Wang Q.; Barras A.; Li M.; Hadjersi T.; Szunerits S.; Boukherroub R. Green Chemistry Approach for the Synthesis of ZnO–Carbon Dots Nanocomposites with Good Photocatalytic Properties under Visible Light. J. Colloid Interface Sci. 2016, 465, 286–294. 10.1016/j.jcis.2015.12.001. 26674245

[ref12] Zhang J.; Zhang X.; Dong S.; Zhou X.; Dong S. N-Doped Carbon Quantum Dots/TiO2 Hybrid Composites with Enhanced Visible Light Driven Photocatalytic Activity toward Dye Wastewater Degradation and Mechanism Insight. J. Photochem. Photobiol., A 2016, 325, 104–110. 10.1016/j.jphotochem.2016.04.012.

[ref13] Sharma S.; Kumar S.; Arumugam S. M.; Elumalai S. Promising Photocatalytic Degradation of Lignin over Carbon Quantum Dots Decorated TiO2 Nanocomposite in Aqueous Condition. Appl. Catal., A 2020, 602, 117730 10.1016/j.apcata.2020.117730.

[ref14] Chen Q.; Wang Y.; Wang Y.; Zhang X.; Duan D.; Fan C. Nitrogen-Doped Carbon Quantum Dots/Ag3PO4 Complex Photocatalysts with Enhanced Visible Light Driven Photocatalytic Activity and Stability. J. Colloid Interface Sci. 2017, 491, 238–245. 10.1016/j.jcis.2016.12.013. 28038396

[ref15] Shen T.; Wang Q.; Guo Z.; Kuang J.; Cao W. Hydrothermal Synthesis of Carbon Quantum Dots Using Different Precursors and Their Combination with TiO2 for Enhanced Photocatalytic Activity. Ceram. Int. 2018, 44, 11828–11834. 10.1016/j.ceramint.2018.03.271.

[ref16] Sharma S.; Umar A.; Sood S.; Mehta S. K.; Kansal S. K. Photoluminescent C-dots: An overview on the recent development in the synthesis, physiochemical properties and potential applications. J. Alloys Compd. 2018, 748, 818–853. 10.1016/j.jallcom.2018.03.001.

[ref17] Chen P.; Wang F.; Chen Z.-F.; Zhang Q.; Su Y.; Shen L.; Yao K.; Liu Y.; Cai Z.; Lv W.; Liu G. Study on the Photocatalytic Mechanism and Detoxicity of Gemfibrozil by a Sunlight-Driven TiO2/Carbon Dots Photocatalyst: The Significant Roles of Reactive Oxygen Species. Appl. Catal., B 2017, 204, 250–259. 10.1016/j.apcatb.2016.11.040.

[ref18] Di J.; Xia J.; Chen X.; Ji M.; Yin S.; Zhang Q.; Li H. Tunable Oxygen Activation Induced by Oxygen Defects in Nitrogen Doped Carbon Quantum Dots for Sustainable Boosting Photocatalysis. Carbon 2017, 114, 601–607. 10.1016/j.carbon.2016.12.030.

[ref19] Huang J.; Wang J.; Hao Z.; Li C.; Wang B.; Qu Y. Fabrication of N-CQDs@W18O49 Heterojunction with Enhanced Charge Separation and Photocatalytic Performance under Full-Spectrum Light Irradiation. Chin. Chem. Lett. 2021, 32, 3180–3184. 10.1016/j.cclet.2021.03.018.

[ref20] Hazarika D.; Karak N. Photocatalytic Degradation of Organic Contaminants under Solar Light Using Carbon Dot/Titanium Dioxide Nanohybrid, Obtained through a Facile Approach. Appl. Surf. Sci. 2016, 376, 276–285. 10.1016/j.apsusc.2016.03.165.

[ref21] Atchudan R.; Edison T. N. J. I.; Perumal S.; Clament Sagaya Selvam N.; Lee Y. R. Green Synthesized Multiple Fluorescent Nitrogen-Doped Carbon Quantum Dots as an Efficient Label-Free Optical Nanoprobe for in Vivo Live-Cell Imaging. J. Photochem. Photobiol., A 2019, 372, 99–107. 10.1016/j.jphotochem.2018.12.011.

[ref22] Yuan H.; Yu J.; Feng S.; Gong Y. Highly Photoluminescent PH-Independent Nitrogen-Doped Carbon Dots for Sensitive and Selective Sensing of p-Nitrophenol. RSC Adv. 2016, 6, 15192–15200. 10.1039/C5RA26870B.

[ref23] He M.; Zhang J.; Wang H.; Kong Y.; Xiao Y.; Xu W. Material and Optical Properties of Fluorescent Carbon Quantum Dots Fabricated from Lemon Juice via Hydrothermal Reaction. Nanoscale Res. Lett. 2018, 13, 175 10.1186/s11671-018-2581-7. 29882047 PMC5992114

[ref24] Jiang G.; Jiang T.; Zhou H.; Yao J.; Kong X. Preparation of N-Doped Carbon Quantum Dots for Highly Sensitive Detection of Dopamine by an Electrochemical Method. RSC Adv. 2015, 5, 9064–9068. 10.1039/C4RA16773B.

[ref25] Shen S.; Chen K.; Wang H.; Fu J. Construction of Carbon Dots-Deposited TiO2 Photocatalysts with Visible-Light-Induced Photocatalytic Activity for the Elimination of Pollutants. Diamond Relat. Mater. 2022, 124, 108896 10.1016/j.diamond.2022.108896.

[ref26] Hamid Abd A.; Adnan Ibrahim O. Synthesis of Carbon Quantum Dot by Electro-Chemical Method and Studying Optical Electrical and Structural Properties. Chem. Methodol. 2022, 6, 823 10.22034/chemm.2022.351559.1575.

[ref27] Wang Y.; Liu Y.; Zhou J.; Yue J.; Xu M.; An B.; Ma C.; Li W.; Liu S. Hydrothermal Synthesis of Nitrogen-Doped Carbon Quantum Dots from Lignin for Formaldehyde Determination. RSC Adv. 2021, 11, 29178–29185. 10.1039/D1RA05370A. 35479568 PMC9040886

[ref28] Architha N.; Ragupathi M.; Shobana C.; Selvankumar T.; Kumar P.; Lee Y. S.; Kalai Selvan R. Microwave-assisted green synthesis of fluorescent carbon quantum dots from Mexican Mint extract for Fe3+ detection and bio-imaging applications. Environ. Res. 2021, 199, 111263 10.1016/j.envres.2021.111263. 33939978

[ref29] Chauhan P.; Dogra S.; Chaudhary S.; Kumar R. Usage of Coconut Coir for Sustainable Production of High-Valued Carbon Dots with Discriminatory Sensing Aptitude toward Metal Ions. Mater. Today Chem. 2020, 16, 100247 10.1016/j.mtchem.2020.100247.

[ref30] Das S. K.; Chakrabarty S.; Gawas R.; Jasuja K. Serendipitous Formation of Photoluminescent Carbon Quantum Dots by Mere Immersion of a Polymer in an Organic Solvent. Carbon Trends 2022, 8, 100183 10.1016/j.cartre.2022.100183.

[ref31] Alamelu K.; Raja V.; Shiamala L.; Jaffar Ali B. M. Biphasic TiO 2 Nanoparticles Decorated Graphene Nanosheets for Visible Light Driven Photocatalytic Degradation of Organic Dyes. Appl. Surf. Sci. 2018, 430, 145–154. 10.1016/j.apsusc.2017.05.054.

[ref32] Joni I. M.; Nulhakim L.; Panatarani C. Characteristics of TiO _2_ Particles Prepared by Simple Solution Method Using TiCl _3_ Precursor. J. Phys.: Conf. Ser. 2018, 1080, 012042 10.1088/1742-6596/1080/1/012042.

[ref33] Johari N. D.; Rosli Z. M.; Juoi J. M.; Yazid S. A. Comparison on the TiO2 Crystalline Phases Deposited via Dip and Spin Coating Using Green Sol–Gel Route. J. Mater. Res. Technol. 2019, 8, 2350–2358. 10.1016/j.jmrt.2019.04.018.

[ref34] Oseghe E. O.; Ofomaja A. E. Facile Microwave Synthesis of Pine Cone Derived C-Doped TiO2 for the Photodegradation of Tetracycline Hydrochloride under Visible-LED Light. J. Environ. Manage. 2018, 223, 860–867. 10.1016/j.jenvman.2018.07.003. 29986335

[ref35] Genc M. T.; Yanalak G.; Arslan G.; Patir I. H. Green Preparation of Carbon Quantum Dots Using Gingko Biloba to Sensitize TiO2 for the Photohydrogen Production. Mater. Sci. Semicond. Process. 2020, 109, 104945 10.1016/j.mssp.2020.104945.

[ref36] Zhao P.; Jin B.; Yan J.; Peng R. Fabrication of Recyclable Reduced Graphene Oxide/Graphitic Carbon Nitride Quantum Dot Aerogel Hybrids with Enhanced Photocatalytic Activity. RSC Adv. 2021, 11, 35147–35155. 10.1039/D1RA06347B. 35493167 PMC9043259

[ref37] Hassan M. E.; Cong L.; Liu G.; Zhu D.; Cai J. Synthesis and Characterization of C-Doped TiO2 Thin Films for Visible-Light-Induced Photocatalytic Degradation of Methyl Orange. Appl. Surf. Sci. 2014, 294, 89–94. 10.1016/j.apsusc.2013.12.069.

[ref38] Liu G.; Jaegermann W.; He J.; Sundström V.; Sun L. XPS and UPS Characterization of the TiO _2_/ZnPcGly Heterointerface: Alignment of Energy Levels. J. Phys. Chem. B 2002, 106, 5814–5819. 10.1021/jp014192b.

[ref39] Chen Y.-H.; Wang B.-K.; Hou W.-C. Graphitic Carbon Nitride Embedded with Graphene Materials towards Photocatalysis of Bisphenol A: The Role of Graphene and Mediation of Superoxide and Singlet Oxygen. Chemosphere 2021, 278, 130334 10.1016/j.chemosphere.2021.130334. 34126674

[ref40] Gong J.; Guo Y.; Lu J.; Cheng Y.; Wang H. TEMPO Oxidized Nanofiber Carbon Quantum Dots/TiO2 Composites with Enhanced Photocatalytic Activity for Degradation of Methylene Blue. Chem. Phys. Lett. 2022, 788, 139297 10.1016/j.cplett.2021.139297.

[ref41] Rani U. A.; Ng L. Y.; Ng C. Y.; Mahmoudi E.; Ng Y.-S.; Mohammad A. W. Sustainable Production of Nitrogen-Doped Carbon Quantum Dots for Photocatalytic Degradation of Methylene Blue and Malachite Green. J. Water Process Eng. 2021, 40, 101816 10.1016/j.jwpe.2020.101816.

[ref42] Sargin I.; Yanalak G.; Arslan G.; Patir I. H. Green Synthesized Carbon Quantum Dots as TiO2 Sensitizers for Photocatalytic Hydrogen Evolution. Int. J. Hydrogen Energy 2019, 44, 21781–21789. 10.1016/j.ijhydene.2019.06.168.

[ref43] Venkateswarlu S.; Viswanath B.; Reddy A. S.; Yoon M. Fungus-Derived Photoluminescent Carbon Nanodots for Ultrasensitive Detection of Hg2+ Ions and Photoinduced Bactericidal Activity. Sens. Actuators, B 2018, 258, 172–183. 10.1016/j.snb.2017.11.044.

[ref44] Yu Y.; Zhou L.; Tang J.; Wu P.; Feng L.; Ge B.; Chen H.; Hu J.; Song S.; Zeng T. Effective Removal of Co(II) and Sr(II) from Radiocative Wastes Using Covalent Triazine Frameworks: Kinetics and Isotherm Studies. Sep. Purif. Technol. 2021, 277, 119633 10.1016/j.seppur.2021.119633.

[ref45] Ma Z.; Yang Z.; Zhang H.; Liu Z. Nitrogen-Doped Microporous Carbon Materials with Uniform Pore Diameters: Design and Applications in CO2 and H2 Adsorption. Microporous Mesoporous Mater. 2020, 296, 109992 10.1016/j.micromeso.2019.109992.

[ref46] Idris M. B.; Sakthivel G.; Devaraj S. Textural Properties Dependent Supercapacitive Performances of Mesoporous Graphitic Carbon Nitride. Mater. Today Energy 2018, 10, 325–335. 10.1016/j.mtener.2018.10.012.

[ref47] Martínez C.; Vilariño S.; Fernández M. I.; Faria J.; L M. C.; Santaballa J. A.; Santaballa J. Mechanism of Degradation of Ketoprofen by Heterogeneous Photocatalysis in Aqueous Solution. Appl. Catal., B 2013, 142–143, 633–646. 10.1016/j.apcatb.2013.05.018.

[ref48] Yu H.; Zhao Y.; Zhou C.; Shang L.; Peng Y.; Cao Y.; Wu L.-Z.; Tung C.-H.; Zhang T. Carbon Quantum Dots/TiO2 Composites for Efficient Photocatalytic Hydrogen Evolution. J. Mater. Chem. A 2014, 2, 3344 10.1039/c3ta14108j.

[ref49] Li M.; Wang M.; Zhu L.; Li Y.; Yan Z.; Shen Z.; Cao X. Facile Microwave Assisted Synthesis of N-Rich Carbon Quantum Dots/Dual-Phase TiO2 Heterostructured Nanocomposites with High Activity in CO2 Photoreduction. Appl. Catal., B 2018, 231, 269–276. 10.1016/j.apcatb.2018.03.027.

[ref50] Wang W.; Ni Y.; Xu Z. One-Step Uniformly Hybrid Carbon Quantum Dots with High-Reactive TiO2 for Photocatalytic Application. J. Alloys Compd. 2015, 622, 303–308. 10.1016/j.jallcom.2014.10.076.

[ref51] Tang Q.-Y.; Chen W.-F.; Lv Y.-R.; Yang S.-Y.; Xu Y.-H. Z-Scheme Hierarchical Cu2S/Bi2WO6 Composites for Improved Photocatalytic Activity of Glyphosate Degradation under Visible Light Irradiation. Sep. Purif. Technol. 2020, 236, 116243 10.1016/j.seppur.2019.116243.

[ref52] Xu L.; Bai X.; Guo L.; Yang S.; Jin P.; Yang L. Facial Fabrication of Carbon Quantum Dots (CDs)-Modified N-TiO2-x Nanocomposite for the Efficient Photoreduction of Cr(VI) under Visible Light. Chem. Eng. J. 2019, 357, 473–486. 10.1016/j.cej.2018.09.172.

[ref53] Ozer M. S.; Eroglu Z.; Yalin A. S.; Kılıç M.; Rothlisberger U.; Metin O. Bismuthene as a Versatile Photocatalyst Operating under Variable Conditions for the Photoredox C H Bond Functionalization. Appl. Catal., B 2022, 304, 120957 10.1016/j.apcatb.2021.120957.

[ref54] Eroglu Z.; Metin O. Internal Interactions within the Complex Type-II Heterojunction of a Graphitic Carbon Nitride/Black Phosphorus Hybrid Decorated with Graphene Quantum Dots: Implications for Photooxidation Performance. ACS Appl. Nano Mater. 2023, 6, 7960–7974. 10.1021/acsanm.3c01187.

[ref55] Xiong J.; Li X.; Huang J.; Gao X.; Chen Z.; Liu J.; Li H.; Kang B.; Yao W.; Zhu Y. CN/RGO@BPQDs High-Low Junctions with Stretching Spatial Charge Separation Ability for Photocatalytic Degradation and H2O2 Production. Appl. Catal., B 2020, 266, 118602 10.1016/j.apcatb.2020.118602.

[ref56] Eroglu Z.; Ozer M. S.; Metin O. Black Phosphorus Quantum Dots/Carbon Nitride-Reduced Graphene Oxide Ternary Heterojunction as a Multifunctional Metal-Free Photocatalyst for Photooxidation Reactions. ACS Sustainable Chem. Eng. 2023, 11, 7560–7572. 10.1021/acssuschemeng.3c01055.

[ref57] Li X.; Luo Q.; Han L.; Deng F.; Yang Y.; Dong F. Enhanced Photocatalytic Degradation and H2 Evolution Performance of NCDs/S-C3N4 S-Scheme Heterojunction Constructed by π-π Conjugate Self-Assembly. J. Mater. Sci. Technol. 2022, 114, 222–232. 10.1016/j.jmst.2021.10.030.

[ref58] Eroglu Z.; Sündü B.; Metin O. Tailoring the Redox Ability of Carbon Nitride Quantum Dots/Reduced Graphene Oxide-Black Phosphorus (CNQDs@rGOBP) Ternary Heterojunctions for Photodegradation of Organic Pollutants. Mater. Today Sustain. 2023, 23, 100418 10.1016/j.mtsust.2023.100418.

[ref59] Xu Q.; Zhang L.; Cheng B.; Fan J.; Yu J. S-Scheme Heterojunction Photocatalyst. Chem 2020, 6, 1543–1559. 10.1016/j.chempr.2020.06.010.

[ref60] Li X.; Kang B.; Dong F.; Zhang Z.; Luo X.; Han L.; Huang J.; Feng Z.; Chen Z.; Xu J.; Peng B.; Wang Z. L. Enhanced Photocatalytic Degradation and H2/H2O2 Production Performance of S-PCN/WO2.72 S-Scheme Heterojunction with Appropriate Surface Oxygen Vacancies. Nano Energy 2021, 81, 105671 10.1016/j.nanoen.2020.105671.

[ref61] Hassani A.; Khataee A.; Karaca S. Photocatalytic Degradation of Ciprofloxacin by Synthesized TiO2 Nanoparticles on Montmorillonite: Effect of Operation Parameters and Artificial Neural Network Modeling. J. Mol. Catal. A: Chem. 2015, 409, 149–161. 10.1016/j.molcata.2015.08.020.

[ref62] Miao Z.; Wang G.; Zhang X.; Dong X. Oxygen Vacancies Modified TiO2/Ti3C2 Derived from MXenes for Enhanced Photocatalytic Degradation of Organic Pollutants: The Crucial Role of Oxygen Vacancy to Schottky Junction. Appl. Surf. Sci. 2020, 528, 146929 10.1016/j.apsusc.2020.146929.

[ref63] Tong S.; Zhou J.; Ding L.; Zhou C.; Liu Y.; Li S.; Meng J.; Zhu S.; Chatterjee S.; Liang F. Preparation of Carbon Quantum Dots/TiO2 Composite and Application for Enhanced Photodegradation of Rhodamine B. Colloids Surf., A 2022, 648, 129342 10.1016/j.colsurfa.2022.129342.

