# Supplementary material for: Boosting Tetracycline Degradation with an S-Scheme Heterojunction of N-Doped Carbon Quantum Dots-Decorated TiO2
Source: ACS Omega. 2023 Jul 12;8(29):26597–609. doi: 10.1021/acsomega.3c03532 (PMC10373195; doi:10.1021/acsomega.3c03532)
Supplement: Supplementary file 1 — ao3c03532_si_001.pdf [file ao3c03532_si_001.pdf]

Boosting Tetracycline Degradation with an S-scheme Heterojunction of N-Doped Carbon Quantum Dots Decorated TiO_2

Melike Karaca[a], Zafer Eroğlu[b], Özkan Açışlı[a], Önder Metin[b,c,*], Semra Karaca[a,**]

[a] Department of Chemistry, Faculty of Science, Atatürk University, 25240 Erzurum, Turkey

[b] Department of Chemistry, College of Sciences, Koç University, 34450 Sarıyer, Istanbul, Turkey

[c] Koç University Surface Science and Technology Center (KUYTAM), 34450 Sarıyer, Istanbul, Turkey

****Corresponding author:** Prof. Semra Karaca, e-mail: skaraca@atauni.edu.tr; semra_karaca@yahoo.com

***Corresponding author:** Prof. Önder Metin, e-mail: ometin@ku.edu.tr; ondermetinnano@gmail.com

1. Materials

Rumex crispus L. plant was collected from Horasan region of Erzurum (in Türkiye), dried, ground and stored for use in experiments. Urea (H_2N-CO-NH_2, 99%) from Merck, hydrochloric acid (HCl, 37%) from Riedel-De-Haën, titanium (IV) ethoxide (TiO_2, >99%), ethanol (99%), and tetracycline hydrochloride (TC, 96%) from Sigma Aldrich purchased taken. Deionized water was used in all experiments. The characteristics and chemical structure of tetracycline (TC) [1] are presented in **Table S1**.

Table S1 Structure and characterization of Tetracycline (TC).

Chemical structure	Molecular formula	λmax (nm)	CAS No
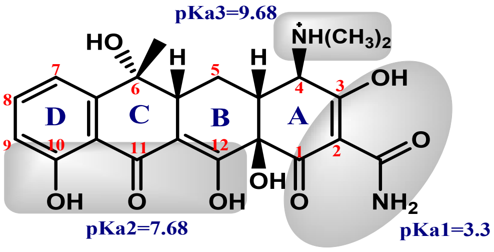	$C_{22}H_{24}N_2O_8$.HCl	357	64-75-5

2. Instrumentation

XRD spectra of the synthesized *N*-CQDs, TiO_2 and *N*-CQDs/TiO_2 samples were collected on a Rigaku Advance Powder X-ray Diffraction meter operating at 30 kV and 30 mA with CuKα radiation in the 2θ of 10-70° range (1.54051 Å). The removal efficiency of tetracycline measurements was performed with Varian Cary 100 UV-VIS Spectrophotometer device (Varian Cary 100, Australia). Fourier transform infrared spectroscopy (FT-IR) was studied with a Tensor 27 Bruker spectrometer (Germany) employing KBr pellets in the 400-4000 cm^{-1} range. The surface morphology and chemical composition of TiO_2, *N*-CQDs and *N*-CQDs/TiO_2 nanocomposites were examined via scanning electron microscope (SEM) by a Zeiss Sigma 300 instrument equipped with an energy dispersive X-ray spectroscopy (EDX). The Brunauer–Emmett–Teller (BET) and Barrett-Joyner-Halenda (BJH) methods allow the analysis of surface area and pore size distributions by nitrogen multilayer adsorption on the Micromeritics 3 Flex instrument, which is based on nitrogen adsorption-desorption isotherms at 77 K. The transmission electron microscopic (TEM) images were acquired with a Hitachi HT7700 TEM (Japan) instrument equipped with EXA- LENS actuated at 120 kV. High resolution scanning electron microscopy (HRSEM) equipped with an energy dispersive X-ray

spectroscopy (EDS) detector (Zeiss Sigma 300 instrument, Germany) was used to take the morphological images of the prepared samples. Photoluminescence (PL) spectra of as-prepared samples were studied on a Shimadzu RF-5301PC spectrofluorophotometer by excitation at 325 nm with a 150 W Xe lamp. The zero-charge point (zpc) of *N*-CQDs/TiO_2 nanocomposite was determined by Malvern Zetasizer Nano ZSP (Malvern Inst. Ltd., UK). The chemical state of photocatalytic material was investigated by X-ray Photo Electron Spectroscopy(XPS, Thermo K-Alpha).To calculate the binding energy adjustment, the C1s peak (284.5 eV) was used as a reference peak. The indium tin oxide (ITO) surface was coated with *N*-CQDs via the drop-casting method.

3. Synthesis of TiO_2

Bare titanium(IV) oxide (TiO_2) was synthesized by using a one-step hydrothermal process. Firstly, 15 mL of concentrated HCl was mixed with 25 mL of distilled water, following 0.3 g of urea was added to the solution, and the final solution was stirred for 10 min. 1.6 mL of titanium(IV) ethoxide was added dropwise into the solution. After a further 15 min of vigorous stirring, the solution was taken to a Teflon-lined stainless-steel autoclave and kept in a temperature-controlled oven at 150°C for 6 h. The autoclave content was then centrifuged at 9000 rpm for 15 min after cooling to ambient temperature. The solid part was separated and dried at 80 °C for 7-8 h. This solid product was calcined in a muffle furnace at 300°C for 2 h.

4. Photocatalytic tetracycline degradation experiments

Photocatalysis of TC was carried out in a quartz cylindrical reactor with a working volume of 500 mL (reactor dimensions 50.0 mm × 250.0 mm, Çalışkan cam, Turkey), and the surface of the reactor was covered with aluminum foil to make more use of the UVA lamp during the experiments. A 16W UV-A lamp (Sylvania, Japan) was used. Batch studies were performed to evaluate the effect of TC concentration, *N*-CQDs/TiO_2 dose, initial solution pH, and presence of various trappers on photocatalytic oxidation. pH adjustments were made using 0.1 N NaOH and 0.1 N HCl, and pH values were monitored using a Mettler Toledo pH meter(China). For each photocatalytic experiment, 500 mL of an aqueous solution including 0.025-0.3 g/L N-CQDs/TiO_2 nanocomposite and 5-30 mg/L TC to the reaction vessel was added. The UV-A lamp was lit at the beginning of each experiment. After stirring for 60 minutes with magnetic stirring to ensure the adsorption-desorption equilibrium, the solution was exposed to UVA irradiation. The solution in the reactor was stirred continuously with a magnetic stirrer.

Approximately 7 mL of solution samples were taken from the suspension at different time intervals, and it was quenched with Na_2SO_3 solution so that the reaction would not exceed the desired time. Then, the samples taken from the reactor at the specified time intervals were centrifuged at 6000 rpm for 10 minutes and the drug concentration in the solution was measured using the Varian Cary 100 UV-Vis spectrophotometer (Australia). In previous measurements, the UV-Visible spectrum of the TC solution was changed from 200 nm to 800 nm. was recorded and the wavelength of the maximum absorbance TC was determined as 357 nm. After the TC concentration versus time was determined, the effects of the parameters on the degradation efficiency were evaluated and the removal efficiency was calculated using following equation:

$$Degradation\ efficiency = \left[\frac{A_0 - A_t}{A_0}\right] x100$$

in which A_0 and A_t exemplify the TC absorbance values for the initial and after t period (min).

5. Catalyst characterization

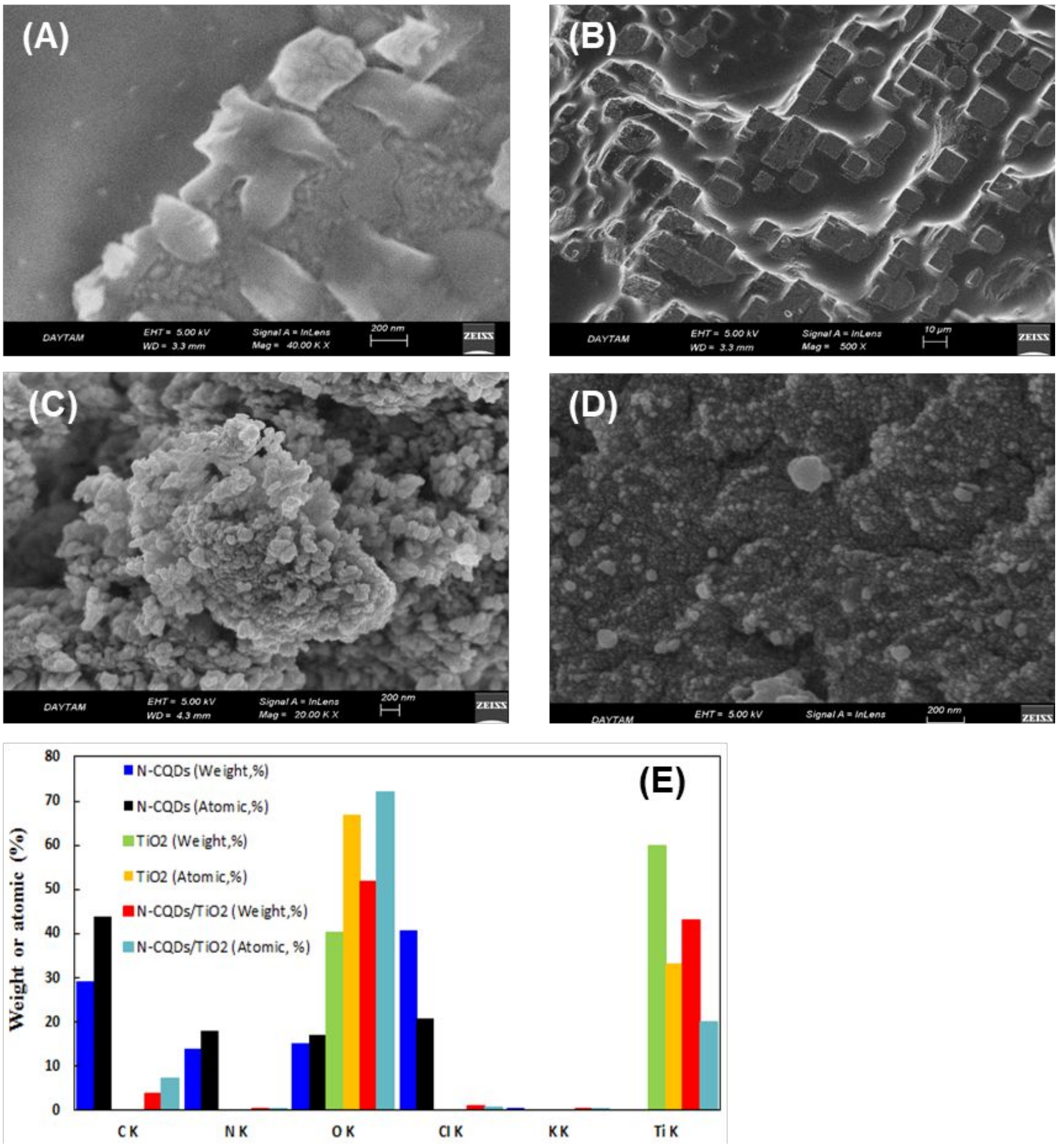

Fig. S1. (A, B)HR-SEM image of pristine *N*-CQDs, **(C)** recorded at different magnifications, **(D)** bareTiO_2, *N*-CQDs/TiO_2 nanocatalysts, and **(E)** EDS analysis results of the prepared materials.

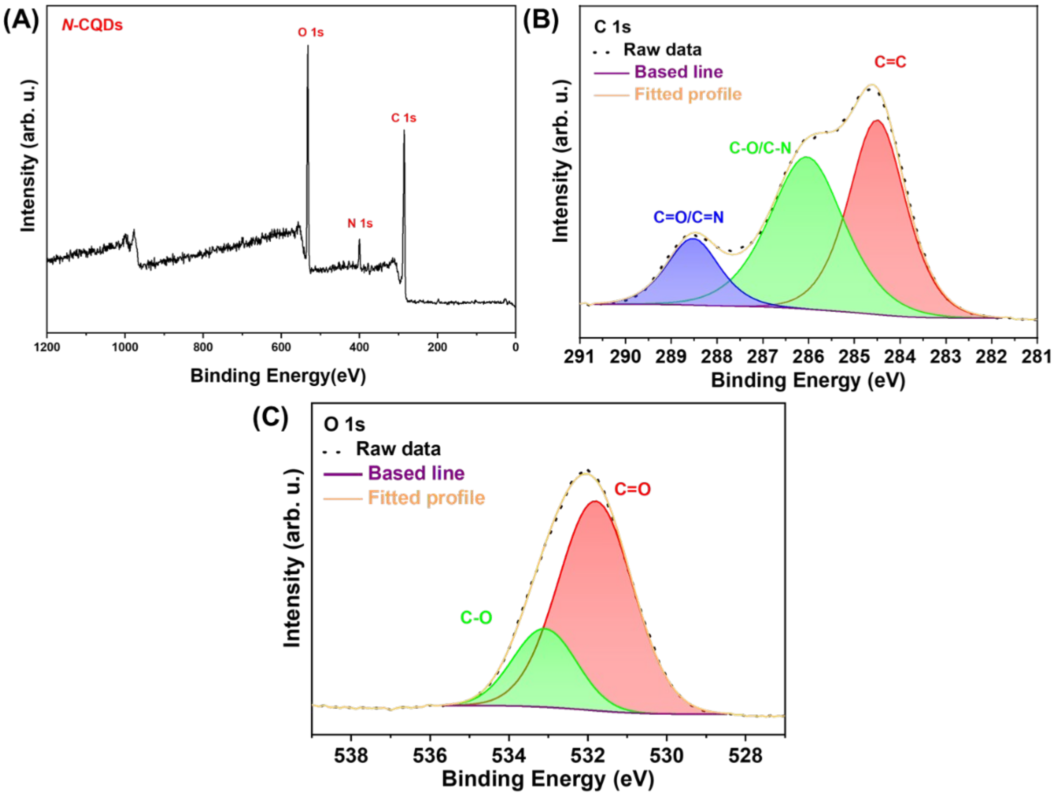

Fig. S2. (A) XPS Survey spectrum **(B)** High-resolution C 1s XPS spectrum **(C)** High-resolution O 1s XPS spectrum of *N*-CQDs.

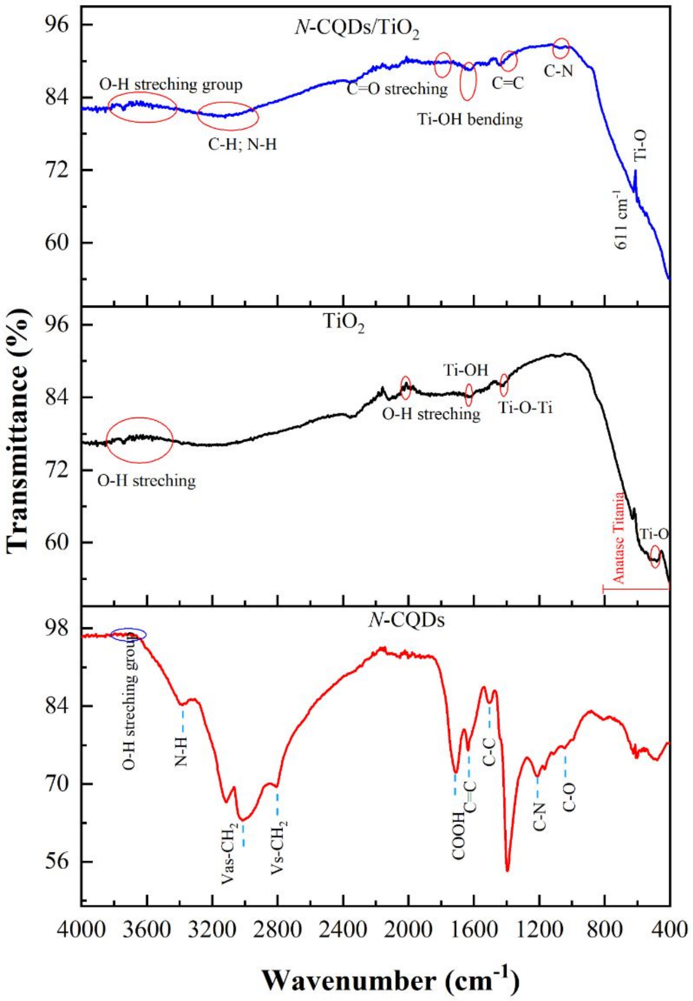

Fig. S3. FTIR spectra of as-prepared samples.

Table S2. Textural properties of the as-synthesized samples.

Catalyst	BET surface area (m^2/g)	Average pore diameter[a] (nm)	Pore volume[b] (cm^3/g)
***N*-CQDs**	0.18	38.79	0.07
**TiO_2**	64.23	11.61	0.21
***N*-CQDs/TiO_2**	161.12	5.01	0.26

[a] Computed by the BJH (desorption) method using N_2 adsorption isotherm.

[b] Found by the BJH method.

6. Photocatalytic tests

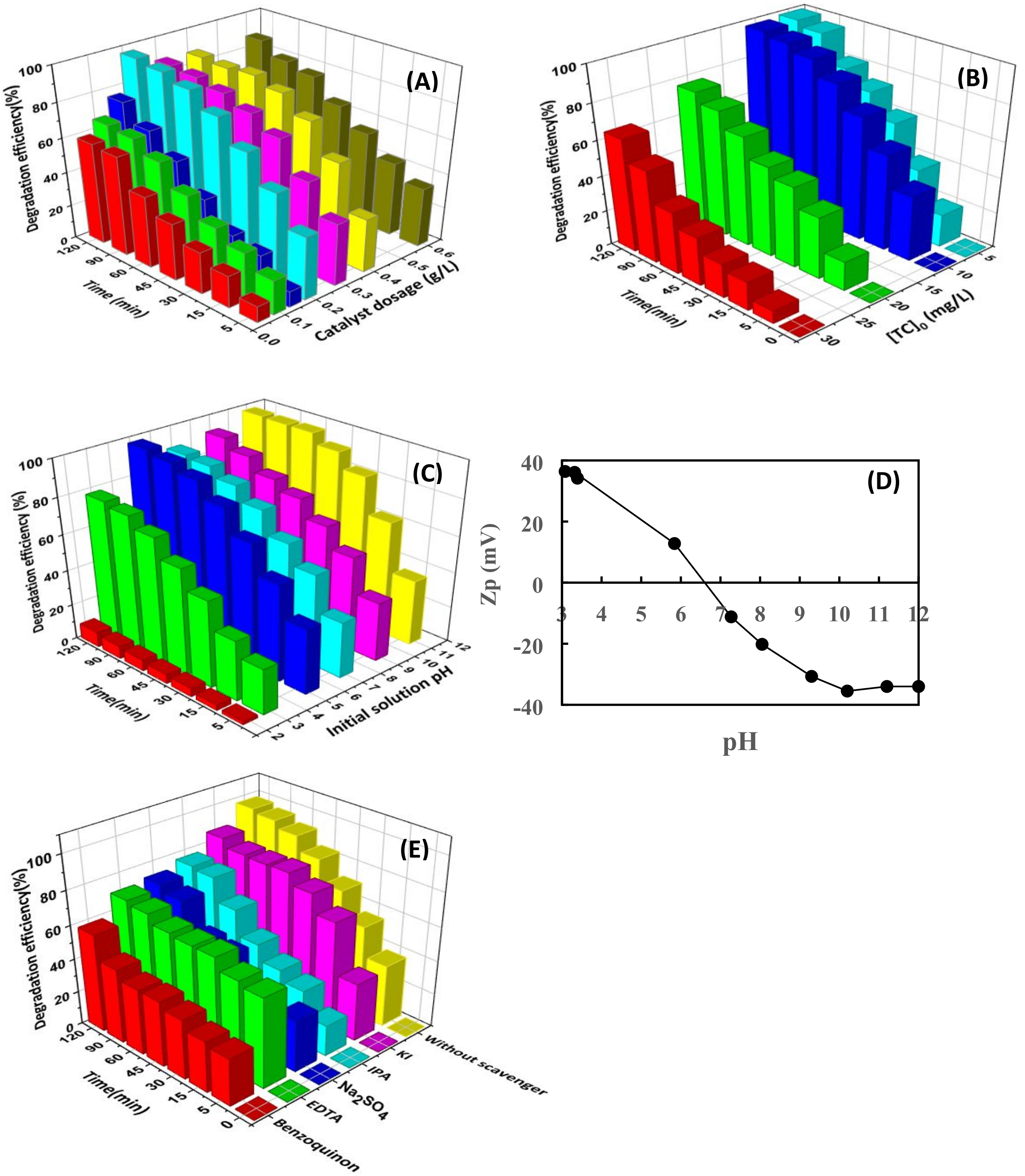

Fig. S4. Impact of some conditions on the percent TC degradation in photocatalytic process. (A) Impact of catalyst concentration. Conditions: $[TC]_0$ = 10 mg/L, and pH = 5.15 (B) Impact of initial concentration. Conditions: $[Catalyst]_0$ = 0.2 g/L, and pH = 5.15. (C) Impact of initial solution pH. Conditions: $[TC]_0$ = 10 mg/L, and $[Catalyst]_0$ = 0.2 g/L(D)Zero point of charge for *N*-CQDs/TiO_2, (E) Impact of scavengers. Conditions: $[TC]_0$ = 10 mg/L, and $[Catalyst]_0$ = 0.2 g/L, $[Scavenger]_0$ = 10 mg/L, and pH = 5.15.

7. Mechanistic studies

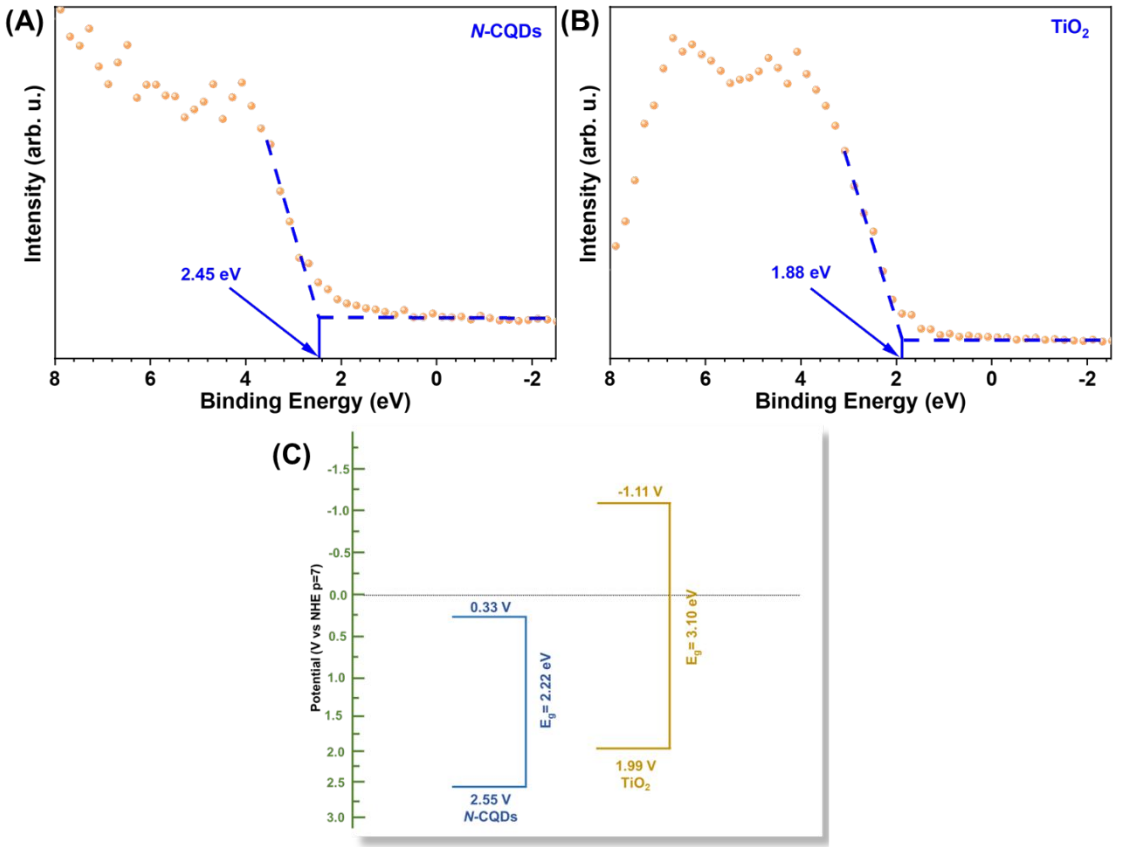

Fig. S5. VB-XPS analyses of **(A)** *N*-CQDs **(B)** TiO_2 **(C)** Band alignments of *N*-CQDs, and TiO_2.

Table S3. Comparison of various catalysts in the photocatalytic oxidation of the tetracycline in water.

Catalyst	TC Conc (mg/L)	Degradation efficiency (%)	Time (min)	Ref.
**SrO-mpg-CN/TiO_2**	10	91.7	180	[2]
**g-C_3N_4/BiOBr**	10	86.1	120	[3]
**$SrTiO_3$/$BaFe_{12}O_{19}$**	15	96.1	120	[4]
**MIP-TiO_2**	20	50.0	90	[5]
**AgCl/ZnO/g-C_3N_4**	10	89.2	50	[6]
**TiO_2@Ti_3C_2**	10	90.7	60	[7]
**TiO_2**	10	95.0	40	[8]
**Ag/$AgIn_5S_8$**	10	95.3	120	[9]
**CDs/g-C_3N_4/MoO_3**	20	88.4	90	[10]
***N*-CQDs/TiO_2**	10	97.7	120	**This study**

REFERENCES

(1) Zhao, Y.; Gu, X.; Gao, S.; Geng, J.; Wang, X. Adsorption of Tetracycline (TC) onto Montmorillonite: Cations and Humic Acid Effects. Geoderma 2012, 183–184, 12–18. https://doi.org/10.1016/j.geoderma.2012.03.004.

(2) Kılıç, D.; Sevim, M.; Eroğlu, Z.; Metin, Ö.; Karaca, S. Strontium Oxide Modified Mesoporous Graphitic Carbon Nitride/Titanium Dioxide Nanocomposites (SrO-Mpg-CN/TiO2) as Efficient Heterojunction Photocatalysts for the Degradation of Tetracycline in Water. *Adv. Powder Technol.* **2021**, *32* (8), 2743–2757. https://doi.org/10.1016/j.apt.2021.05.043.

(3) Shi, Z.; Zhang, Y.; Shen, X.; Duoerkun, G.; Zhu, B.; Zhang, L.; Li, M.; Chen, Z. Fabrication of G-C3N4/BiOBr Heterojunctions on Carbon Fibers as Weaveable Photocatalyst for Degrading Tetracycline Hydrochloride under Visible Light. *Chem. Eng. J.* **2020**, *386*, 124010. https://doi.org/10.1016/j.cej.2020.124010.

(4) Feng, S.; Xie, T.; Wang, J.; Yang, J.; Kong, D.; Liu, C.; Chen, S.; Yang, F.; Pan, M.; Yang, J.; Du, H.; Chen, H. Photocatalytic Activation of PMS over Magnetic Heterojunction Photocatalyst SrTiO3/BaFe12O19 for Tetracycline Ultrafast Degradation. *Chem. Eng. J.* **2023**, 143900. https://doi.org/10.1016/j.cej.2023.143900.

(5) Wang, H.; Wu, X.; Zhao, H.; Quan, X. Enhanced Photocatalytic Degradation of Tetracycline Hydrochloride by Molecular Imprinted Film Modified TiO2 Nanotubes. *Chin. Sci. Bull.* **2012**, *57* (6), 601–605. https://doi.org/10.1007/s11434-011-4897-x.

(6) Ding, C.; Zhu, Q.; Yang, B.; Petropoulos, E.; Xue, L.; Feng, Y.; He, S.; Yang, L. Efficient Photocatalysis of Tetracycline Hydrochloride (TC-HCl) from Pharmaceutical Wastewater Using AgCl/ZnO/g-C3N4 Composite under Visible Light: Process and Mechanisms. *J. Environ. Sci.* **2023**, *126*, 249–262. https://doi.org/10.1016/j.jes.2022.02.032.

(7) Bui, H. T.; Van Thuan, D.; Thi Huong, P.; Nguyen, K. D.; Nguyen, M. V.; Chu, T. T. H.; Le, Q. V.; Jitae, K.; Devanesan, S.; AlSalhi, M. S.; Nguyen, T. L. Enhanced Photocatalytic H2 Evolution and Photodegradation of Antibiotic Tetracycline in Wastewater by TiO2@Ti3C2. *Int. J. Hydrog. Energy* **2022**. https://doi.org/10.1016/j.ijhydene.2022.07.128.

(8) Zhu, X.-D.; Wang, Y.-J.; Sun, R.-J.; Zhou, D.-M. Photocatalytic Degradation of Tetracycline in Aqueous Solution by Nanosized TiO2. *Chemosphere* **2013**, *92* (8), 925–932. https://doi.org/10.1016/j.chemosphere.2013.02.066.

(9) Deng, F.; Zhao, L.; Luo, X.; Luo, S.; Dionysiou, D. D. Highly Efficient Visible-Light Photocatalytic Performance of Ag/AgIn5S8 for Degradation of Tetracycline Hydrochloride and Treatment of Real Pharmaceutical Industry Wastewater. *Chem. Eng. J.* **2018**, *333*, 423–433. https://doi.org/10.1016/j.cej.2017.09.022.

(10) Xie, Z.; Feng, Y.; Wang, F.; Chen, D.; Zhang, Q.; Zeng, Y.; Lv, W.; Liu, G. Construction of Carbon Dots Modified MoO3/g-C3N4 Z-Scheme Photocatalyst with Enhanced Visible-Light Photocatalytic Activity for the Degradation of Tetracycline. *Appl. Catal. B Environ.* **2018**, *229*, 96–104. https://doi.org/10.1016/j.apcatb.2018.02.011.
